# Local Adaptation and the Evolution of Genome Architecture in Threespine Stickleback

**DOI:** 10.1093/gbe/evac075

**Published:** 2022-05-20

**Authors:** Qiushi Li, Dorothea Lindtke, Carlos Rodríguez-Ramírez, Ryo Kakioka, Hiroshi Takahashi, Atsushi Toyoda, Jun Kitano, Rachel L. Ehrlich, Joshua Chang Mell, Sam Yeaman

**Affiliations:** Department of Biological Sciences, University of Calgary, 2500 University Drive NW, Calgary, Canada T2N 1N4; Department of Biological Sciences, University of Calgary, 2500 University Drive NW, Calgary, Canada T2N 1N4; Division of Evolutionary Ecology, Institute of Ecology and Evolution, University of Bern, Bern, Switzerland; Tropical Biosphere Research Center, University of the Ryukyus, Nishihara, Nakagami-gun, Okinawa 903-0213, Japan; National Fisheries University, 2-7-1 Nagata-honmachi, Shimonoseki, Yamaguchi 759-6595, Japan; Comparative Genomics Laboratory, National Institute of Genetics, Mishima, Shizuoka 411-8540, Japan; Ecological Genetics Laboratory, National Institute of Genetics, Mishima, Shizuoka 411-8540, Japan; Department of Microbiology & Immunology, Drexel University College of Medicine, Philadelphia 19102, PA, USA; Department of Microbiology & Immunology, Drexel University College of Medicine, Philadelphia 19102, PA, USA; Department of Biological Sciences, University of Calgary, 2500 University Drive NW, Calgary, Canada T2N 1N4

**Keywords:** genome evolution, chromosomal rearrangement, local adaptation, transposable element, gene flow

## Abstract

Theory predicts that local adaptation should favor the evolution of a concentrated genetic architecture, where the alleles driving adaptive divergence are tightly clustered on chromosomes. Adaptation to marine versus freshwater environments in threespine stickleback has resulted in an architecture that seems consistent with this prediction: divergence among populations is mainly driven by a few genomic regions harboring multiple quantitative trait loci for environmentally adapted traits, as well as candidate genes with well-established phenotypic effects. One theory for the evolution of these “genomic islands” is that rearrangements remodel the genome to bring causal loci into tight proximity, but this has not been studied explicitly. We tested this theory using synteny analysis to identify micro- and macro-rearrangements in the stickleback genome and assess their potential involvement in the evolution of genomic islands. To identify rearrangements, we conducted a de novo assembly of the closely related tubesnout (*Aulorhyncus flavidus*) genome and compared this to the genomes of threespine stickleback and two other closely related species. We found that small rearrangements, within-chromosome duplications, and lineage-specific genes (LSGs) were enriched around genomic islands, and that all three chromosomes harboring large genomic islands have experienced macro-rearrangements. We also found that duplicates and micro-rearrangements are 9.9× and 2.9× more likely to involve genes differentially expressed between marine and freshwater genotypes. While not conclusive, these results are consistent with the explanation that strong divergent selection on candidate genes drove the recruitment of rearrangements to yield clusters of locally adaptive loci.

**Significance**

The architecture of the genome can evolve through chromosomal rearrangements, duplications, and deletions, but this is thought to be a largely random process, with selection purging deleterious changes. Here, we explore whether such changes tend to evolve most rapidly in regions of the genome involved in local adaptation to freshwater versus saltwater in the threespine stickleback. We find enrichment of several types of rearrangement in these regions, which often involve movement or duplication of genes that are differentially expressed in freshwater- versus saltwater-adapted genotypes. As clustering of causal loci is theoretically favored under local adaptation, clustering of these rearrangements suggests that evolution may be actively reshaping the genome to favor a higher-fitness architecture.

## Introduction

Many species inhabit heterogeneous environments where spatial differences in the direction of natural selection drive adaptation to the local environment (Hedrick et al. 1976; Hereford 2009). When migration rate among populations is sufficiently high, an evolutionary tension develops with divergent selection that can profoundly affect the genetic architecture of local adaptation. Because weakly selected alleles are susceptible to “swamping” by migration under these conditions (Haldane 1930; Lenormand 2002), there is a general advantage for alleles with larger effects and/or tightly linked clusters of alleles with smaller effects (Yeaman and Otto 2011; Yeaman and Whitlock 2011). This advantage of such “concentrated” genetic architectures is expected to favor the evolution of clustering of causal alleles (Feder et al. 2012; Via 2012) which can occur via three broad types of mechanism: (1) differential probability of establishment, persistence time, or competition favoring alleles that are more tightly linked (Yeaman and Whitlock 2011; Aeschbacher and Buerger 2014; Yeaman et al. 2016); (2) modifiers reducing the rate of recombination between existing loosely linked alleles (e.g., by the establishment of an inversion capturing the alleles, Noor et al. 2001; Rieseberg 2001; Kirkpatrick and Barton 2006); (3) fixation of a chromosomal rearrangement moving a causal locus into close proximity with other causal loci (Yeaman 2013; Guerrero and Kirkpatrick 2014).

While evidence in some species seems consistent with the concentrated architectures hypothesis, much remains unclear about which mechanisms drive their evolution. Empirical work has revealed a wide range of patterns in the genomic landscape of differentiation underlying local adaptation, with some studies finding large clusters of loci that are highly differentiated between populations (“genomic islands”), but others finding little evidence for such patterns (Nosil et al. 2009; Cruickshank and Hahn 2014; Yeaman 2022). Unfortunately, when genomic islands are found it is typically unclear which loci within them are selected versus neutral, so it is difficult to infer if this is evidence for clustering of causal loci. Furthermore, in many cases, genomic islands could also be explained as artifacts arising from linkage and background or positive selection (Noor and Bennett 2009; Cruickshank and Hahn 2014; Booker et al. 2021). If genomic islands do in fact represent concentrated architectures, it is particularly interesting to know whether rearrangements contributed to their evolution, because this constitutes a durable change in the architecture of the genome. The other mechanisms of architecture evolution (1 and 2) depend on the segregation of alleles or inversions, which could be lost following an extreme population bottleneck.

Clear evidence of clustering has been found for the genes involved in secondary metabolic pathways in many plants (Nützmann and Osbourn 2014; Slot and Gluck-Thaler 2019), but it is unclear whether such clustering has evolved to reduce recombination or for some other more proximate benefit, such as coordination of gene expression or translation. Some fascinating examples of “supergene” architectures with tightly clustered alleles have been found in species experiencing local adaptation or negative frequency-dependent selection within populations (Schwander et al. 2014; Thompson and Jiggins 2014; Charlesworth 2016), such as in the social chromosomes in ants (Wang et al. 2013; Purcell et al. 2014), wing-color pattern in Heliconius butterflies (Joron et al. 2011), floral architecture in petunia (Hermann et al. 2013), and coloration in stick insects (Villoutreix et al. 2020). However, in most cases it is unclear whether such supergenes evolved through allelic replacement (mechanism 1, above) or rearrangement of underlying loci (mechanism 3; Charlesworth and Charlesworth 1975), and in many cases these supergenes are also associated with inversions.

Here, we approach this question from the other direction, beginning with regions of the genome known to be involved in local adaptation, and asking whether such regions have experienced more rapid evolution in genome organization and architecture. While most rearrangements likely evolve under the balance between mutation, drift, and purifying selection, an increased occurrence in the genomic regions involved in local adaptation would be unlikely to occur under this null model. By contrast, if local adaptation has favored the fixation of rearrangements to create clusters of causal loci with increased linkage (Yeaman 2013), we would expect to see an enrichment of such events in genomic regions driving local adaptation. We also study changes in macro-scale chromosomal architecture, as fusions can bring together larger regions of the genome harboring multiple genomic islands, due to a similar advantage for local adaptation (Guerrero and Kirkpatrick 2014).

We explore this question by studying the evolution of genome architecture in threespine stickleback (*Gasterosteus aculeatus*), a model species for the study of ecological adaptation. Extensive study has revealed regions of the genome that are disproportionately involved in local adaptation for freshwater versus saltwater environments, harboring large numbers of linked quantitative trait loci (QTL) for a range of ecologically important traits (Miller et al. 2014; Peichel and Marques 2017; Erickson et al. 2018) that tend to cooccur with genomic islands of differentiation (Hohenlohe et al. 2010; Jones, Grabherr, et al. 2012; Jones, Rajaraman, et al. 2012; Samuk et al. 2017; Kingman et al. 2021). Importantly, threespine stickleback has been undergoing repeated bouts of local adaptation to freshwater through many cycles of extirpation and recolonization over millions of years (Bell and Foster 1994; Schluter and Conte 2009; Nelson and Cresko 2018), while outgroup species such as tubesnout and seabass are obligately marine. While it is unclear exactly when this lineage began colonizing freshwater, other species in the stickleback clade also inhabit both marine and brackish or freshwater environments (Kawahara et al. 2009), indicating that this is an old adaptive strategy. Given that theory shows that genome evolution in response to these evolutionary pressures is likely to be slow (Yeaman 2013), it is important to test this theory in a clade that has experienced a prolonged evolutionary history of inhabiting a strongly heterogenous selection environment, making the threespine stickleback a strong candidate.

**Fig. 1.**
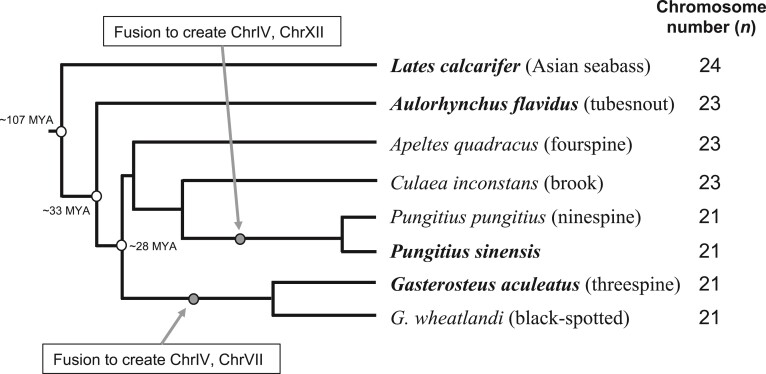
Phylogeny of the stickleback and closely related species. Chromosome numbers are derived from Urton et al. (2011) and Vij et al. (2016), and the current study. Divergence times were estimated by taking the median across a number of studies using Timetree (Kumar et al. 2017), which places the *Gasterosteus* and *Pungitius-Apeltes* split at ∼27.8 Ma (confidence interval, CI = 19.0–32.1 Ma), the stickleback and tubesnout split at ∼33 Ma (CI = 25–43 Ma), and the split with Asian seabass at ∼107 Ma (CI = 94–115 Ma). Redrawn based on Kawahara et al. (2009); a more recent phylogeny based on genome-wide data also groups both *Apeltes quadracus* and *Culaea inconstans* with the *Pungitius* clade with 100% bootstrap support (fig. 2*A* in Guo et al. 2019); branch lengths are not drawn to scale.

Previous studies have revealed changes in karyotype within the stickleback clade (Ross et al. 2009; Urton et al. 2011; Rastas et al. 2016; Varadharajan et al. 2019), indicating some kinds of macro-rearrangements. Despite being less closely related (fig. 1), the *Gasterosteus* and *Pungitius* sticklebacks are more similar in their karyotype than the other close relatives of *Pungitius* (fourspine and brook stickleback). Both *Gasterosteus* and *Pungitius* have *n* = 21 chromosomes (compared with *n* = 23 in fourspine and brook) and have syntenic arrangements for ChrIV, which is homologous to two smaller chromosomes in fourspine stickleback (Ross et al. 2009; Urton et al. 2011; Rastas et al. 2016; Varadharajan et al. 2019). *Gasterosteus* and *Pungitius* differ in ChrVII: in *Gasterosteus* it is homologous to two smaller chromosomes in fourspine stickleback, but in *Pungitius* one of these smaller chromosomes has fused with the chromosome ancestral to ChrXII in threespine (Urton et al. 2011; Rastas et al. 2016; Varadharajan et al. 2019). Thus, it is unclear how karyotype has evolved within this group of species and which architecture more closely resembles the ancestral form, although it is evident that at least two of the three chromosomes most commonly involved in local adaptation have experienced some large-scale rearrangements.

To study the interplay between genome evolution and local adaptation in threespine stickleback, we reconstruct the history of macro- and micro-rearrangements by comparing the genomic position of orthologs among closely related species. As this requires comparison with an outgroup species, we construct the first chromosome-scale de novo genome assembly of tubesnout (*Aulorhynchus flavidus*), a closely related and obligately marine outgroup of the stickleback clade, and compare this to the recently published assembly of another stickleback (*Pungitius sinensis*; Yamasaki et al. 2020). We use Asian seabass (*Lates calcarifer*; Vij et al. 2016) and additional outgroup species to improve orthology reconstruction and identify whether putative rearrangements happened in the tubesnout or stickleback lineage. For small-scale changes in genome architecture, we characterize three types of events, which we collectively refer to as Micro Genome Evolution Events (MGEEs): within-chromosome gene duplications, interchromosomal rearrangement of one or more adjacent genes, and lineage-specific genes (LSGs) suggestive of de novo gene birth. We then test whether these MGEEs tend to be enriched within and around genomic islands for marine versus freshwater divergence identified by Kingman et al. (2021). While de novo gene birth is not a rearrangement, if it results in a novel adaptive function and occurs in a beneficial linkage relationship to other locally adapted loci in a genomic island, this would favor the recruitment of LSGs within genomic islands above the background rate. Our analysis of the distribution of both macro and micro-rearrangements placed *a priori* focus on chromosomes IV, VII, and XXI, as numerous lines of evidence from QTL studies and genome scans show they tend to be overrepresented in their contributions to marine versus freshwater local adaptation (Hohenlohe et al. 2010; Jones, Grabherr, et al. 2012; Jones, Rajaraman, et al. 2012; Miller et al. 2014; Peichel and Marques 2017; Samuk et al. 2017; Erickson et al. 2018), and harbor a number of candidate genes identified by fine-scale mapping, including *Eda* (Colosimo et al. 2005), *Msx2a* (Howes et al. 2017), *Wnt7b* (Jones, Grabherr, et al. 2012; Jones, Rajaraman, et al. 2012), *Pitx1* (Shapiro et al. 2004), *Tfap2a* (Erickson et al. 2018), and *Bmp6* (Cleves et al. 2014; see supplementary table S1 and materials, Supplementary Material online for further details about methods development).

## Results

### First Draft De Novo Assembly of the Tubesnout Genome

The estimated genome size of the male tubesnout used in this study was 468.9 Mb based on the 16 k-mer frequency counting result using Illumina sequence (supplementary fig. S1, Supplementary Material online). The kmer-individual heterozygous ratio is about 0.032, indicating the high heterozygosity of the sample, and 12.18% of the genome was categorized as the repetitive content. We used Pacbio (RS II) long reads (50.3 Gb, >100× coverage) generated from a 20 kb insert-size SMRTbell library for the contig-level assembly to ensure accuracy. We obtained 1,118 phased haplotigs with a total length of 488.5 Mb and N50 length of 2.2 Mb, which was subsequently polished with 226× Illumina short reads and used as the input for Hi-C scaffolding. Mis-joins and duplicates of the haplotigs were solved based on the chromatin conformation information captured from the same individual. Finally, contigs totaling 445.6 Mb, accounting for 97.1% of the total 458.8 Mb assembled genome sequences, were clustered into 23 chromosome-scale scaffolds in the Hi-C scaffolding step (supplementary fig. S2, Supplementary Material online). BUSCO assessment with the Actinopterygii database composed of 4,584 BUSCOs revealed a completeness summary of complete orthologs: 94.3% (single-copy: 92.1%, duplicated: 2.2%), fragmented orthologs: 2.6%, missing orthologs: 3.1%. The 23 chromosome scaffolds show great consistency in contiguity when compared with the 10 longest reads (>100 kb) assembled following the orthogonal linked-reads strategy (supplementary fig. S3, Supplementary Material online). All these data demonstrate a high-quality genome assembly of the tubesnout.

### Macro-Rearrangements in Stickleback

**Fig. 2.**
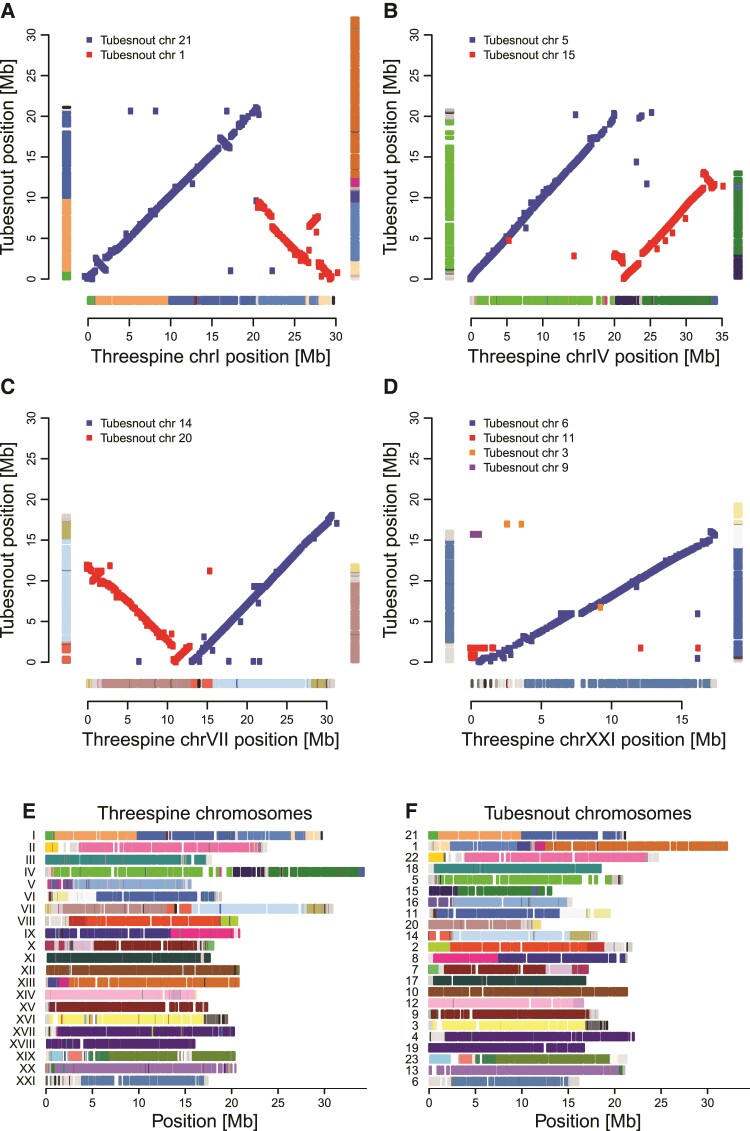
Patterns of synteny between threespine stickleback and tubesnout. Panels (*A–D*) show dot plots based on the positions of orthologs reconstructed by method 2, while colored bars on the sides (*A–D*) and in (*E* and *F*) indicate patterns of synteny identified by method 1, with the largest 47 CARs plotted in color and the remaining 202 minor CARs plotted in grayscale. Note that stickleback ChrXIII is homologous to part of tubesnout chromosome 1.

**Fig. 3.**
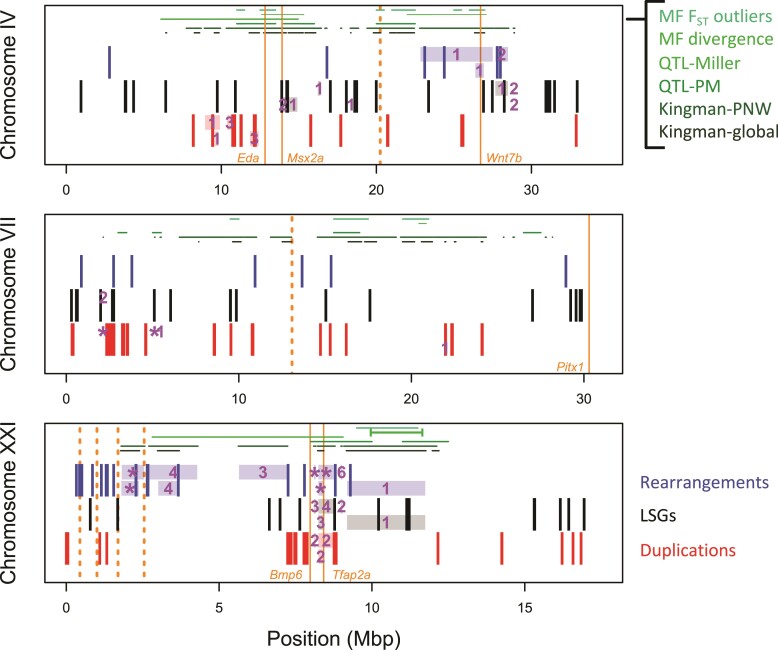
Chromosomal distribution of three types of MGEE: micro-rearrangements (blue), lineage-specific genes (LSGs; black), and duplications (red) across the three focal chromosomes commonly involved in local adaptation. Shaded rectangles indicate regions that are significantly enriched for each type of MGEE (*P* < 0.05) around the Kingman PNW (above) and global (below) sets of genomic islands. Purple numbers indicate how many of the seven window sizes were found to be significant (*P* < 0.05), with “*” indicating an island that was significant following FDR correction across all islands tested (*q* < 0.05) for at least one window size. The locations of candidate genes for local adaptation are shown with solid orange lines; orange dashed lines indicate the approximate location of breakpoints for ancestral macro-rearrangements. Lines along the top of each panel indicate positions where: mean *F*_ST_ between marine–freshwater populations from Samuk et al. (2017) falls in the top 5% of the distribution; extreme marine–freshwater divergence identified by Jones, Grabherr, et al. (2012) with the inversion on ChrXXI identified as a bounded line); QTL identified by Miller et al. (2014); number of QTL from the meta-analysis of Peichel and Marques (2017) falls into the top 5% of the distribution (dark green), and the PNW and global sets of genomic islands from Kingman et al (2021).

Both methods (1 and 2; see Materials and Methods) for identifying rearrangements revealed that both chromosomes IV and VII had undergone fusions somewhere on the threespine stickleback lineage, as the homologous regions in both seabass and tubesnout are present as two separate chromosomes in each case (figs. 1 and 2, supplementary figs. S4–S6, Supplementary Material online). Consistent with the prediction of these macro-rearrangements being driven by an advantage for local adaptation, the fusions creating both chromosomes IV and VII involved regions of the genome harboring genomic islands strongly implicated in local adaptation in threespine stickleback (fig. 3). These regions would have been on separate chromosomes and therefore freely recombining before the ancestral fusion. These results show that fusions of the same two parent chromosomes independently created ChrIV in both *Pungitius* and *Gasterosteus*, which would be very unlikely to happen by chance. Previous data showed that ChrIV is syntenic but not collinear in these species (Rastas et al. 2016; Varadharajan et al. 2019) and our comparison with tubesnout and seabass shows that the nonfused architecture of this chromosome was most likely ancestral, and it is also presumably shared with fourspine stickleback, which has the same number of chromosomes as tubesnout (fig. 1; Urton et al. 2011). Another analysis of the genomes of several stickleback species, including a *de novo* fourspine stickleback genome assembly, has come to similar conclusions about their macro-evolutionary history (Liu, Chen, et al. 2021; Liu, Roesti, et al. 2022). Also on the threespine stickleback lineage, our reconstructions show that chromosome I experienced a large translocation (involving homologs to tubesnout chromosomes 1 and 21; fig. 2) and chromosome XXI experienced a series of complex rearrangements at one end involving three tubesnout homologs (supplementary results and fig. S6, Supplementary Material online), while the other 17 chromosomes are broadly conserved in their synteny between stickleback and tubesnout (supplementary table S2, Supplementary Material online).

Taken together, these results show that all three of the chromosomes most commonly involved in local adaptation in threespine stickleback have undergone macro-scale rearrangements in the threespine stickleback lineage, but that only one other chromosome has done so. If the chance of each of the 21 stickleback chromosomes undergoing such macro-rearrangement is equal, the probability that all three chromosomes with pronounced genomic islands experienced rearrangement is *P* = 0.003, given four random draws from 21 without replacement. Alternatively, if the chance of rearrangement is proportional to chromosome length in threespine stickleback, then this probability is *P* = 0.0064 (by 100,000 random draws).

### Characteristics of MGEEs

We observed a total of 154 micro-rearrangements, but in some cases we could not conclusively determine whether they had occurred in the tubesnout or threespine stickleback lineage. If all of these occurred on the stickleback lineage, the long-term rate of occurrence of such events would be ∼4.7/Myr, given the divergence time of 33 Myr (Kumar et al. 2017), although this might be overestimated by up to ∼2× if some events in tubesnout were mis-attributed to stickleback. We observed a total of 288 LSGs common to both threespine stickleback and *P. sinensis* (70 of which were high confidence). Given estimated divergence times of ∼27.8 Ma between threespine stickleback and *P. sinensis* and 33 Ma between the sticklebacks and tubesnout, this suggests a burst of LSGs in the early stages of stickleback evolution (288 over ∼4.2 Myr). We observed 248 duplications in stickleback not found in tubesnout, which would correspond to a rate of 7.5/Myr. The size of the genes involved in micro-rearrangements (mean = 831.1 bp), LSGs (472.4 bp), and duplications (1,008.3 bp) tended to be significantly smaller than for genes that have not undergone such events (mean = 1,582.9 bp; Wilcoxon rank sum test, *P* < 10^−15^ in all cases). We were able to annotate 238 out of the 248 duplicated genes and 128 out of 182 of the rearranged genes. We conducted a test of gene ontology (GO) enrichment in these genes and interestingly found a significant enrichment of genes related to olfactory receptors and the hemoglobin complex on the duplicated genes, and an enrichment of genes related to the dynein complex on the rearranged genes (supplementary table S4, Supplementary Material online).

### Genomic Distribution of MGEEs

In order to test for enrichment of MGEEs around genomic islands, it was first necessary to characterize the broad-scale patterns in their distribution to develop null models for enrichment testing. In chromosomes without a history of macro-rearrangement, the density of rearrangements and LSGs tended to be higher towards the ends of chromosomes, whereas duplications exhibited a less consistent pattern with reduced density near the ends of chromosomes (supplementary fig. S7, Supplementary Material online). We found no consistent differences in patterns of MGEE occurrence between acro-, meta-, telo-centric chromosomes (supplementary fig. S8, Supplementary Material online), and these patterns did not consistently covary with gene density (supplementary fig. S7, Supplementary Material online).

**Fig. 4.**
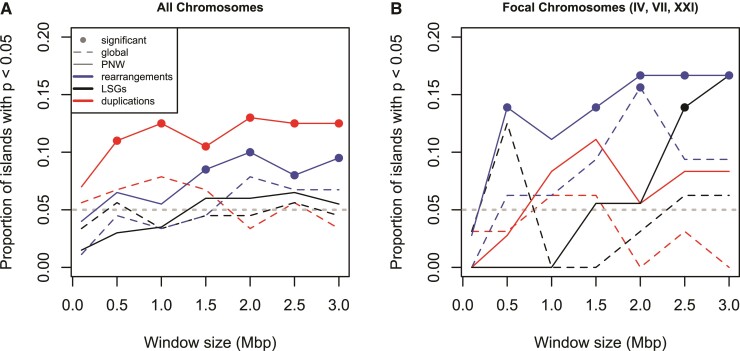
The proportion of genomic islands in the PNW and global sets of “ecopeaks” from Kingman et al. (2021) with significant enrichment of different types of MGEE. Enrichment analysis was conducted using the double-adj model within windows of various sizes around each genomic island. Results are shown for the 200 (PNW) and 89 (global) genomic islands across all chromosomes (*A*) and the 36 (PNW) and 32 (global) islands within the focal chromosomes most commonly involved in local adaptation to marine versus freshwater (*B*). Significance indicated by the filled dots occurs when the number of windows with *P* < 0.05 exceeds the 95th percentile of a binomial distribution, with the null expectation of 5% indicated by a horizontal dashed gray line.

To test for enrichment around genomic islands, we compared observed counts of MGEEs to expectations under a null model based on their respective density patterns (supplementary fig. S7, Supplementary Material online), applied separately on either side of the ancestral breakpoint in chromosomes that had undergone macro-rearrangement (the “double-adj” model, supplementary fig. S9, Supplementary Material online). We tested enrichment of each type of MGEE in windows up to 3 Mbp around the genomic islands identified by Kingman et al. (2021) for both the Pacific NorthWest (PNW) and global regions. At the whole genome scale, we found that both duplications and rearrangements were enriched (*P* < 0.05) in significantly more genomic islands than expected (i.e., ≫5% of cases, by a binomial test) for the PNW set, but not for the global set (fig. 4*A*). Similar patterns of enrichment were found for rearrangements around genomic islands in the focal chromosomes (IV, VII, and XXI), but duplications showed somewhat reduced enrichment that was nonsignificant, perhaps due to the lower power associated with a smaller number of genomic islands (fig. 4*B*). LSGs showed less consistent patterns that were only significantly enriched at a few window sizes (fig. 4). These patterns were largely robust to the null model with similar results found using the gene density, flat, and single-adj models, with the exception of a loss of significance for rearrangements in the flat model (supplementary fig. S10, Supplementary Material online).

Examining patterns within the focal chromosomes, we found particularly strong signatures of enrichment for all three types MGEE in genomic islands near *Bmp6* and *Tfap2a* on ChrXXI (fig. 3), which are genes known to be involved with tooth gain and craniofacial architecture in stickleback (Cleves et al. 2014; Erickson et al. 2018). Micro-rearrangements were also significantly enriched in genomic islands near the complex macro-rearrangements on Chr XXI. Less pronounced signatures of enrichment were found in genomic islands on chromosomes IV and VII, with the strongest of these being for duplications in genomic islands near the *Eda* and *Msx2a* genes, which are involved with local adaptation to freshwater (Colosimo et al. 2005; Howes et al. 2017; Schluter et al. 2021). The degree of significance of these patterns of enrichment varies with the choice of null model, but the broad patterns remain significant regardless (supplementary figs. S11–S13, Supplementary Material online), so the choice of density model does not seem to be driving our results. Similar patterns of enrichment were also found applying this method to test enrichment around the main candidate genes on the focal chromosomes (supplementary table S4, Supplementary Material online). Examining patterns of MGEE distribution irrespective of genomic islands, there was significant enrichment of duplications on chromosomes XIX, X, and XI, of rearrangements on XXI and X, and of LSGs on XII (supplementary fig. S14, Supplementary Material online). Thus, the above patterns of enrichment within genomic islands on the focal chromosomes do not arise from an overall higher rate of MGEEs on these chromosomes.

It is possible that the above patterns of enrichment of MGEEs around genomic islands were driven by an increased rate of occurrence near macro-rearrangement breakpoints, if genomic islands happen to also be close to these breakpoints. To test this alternative hypothesis, we applied the same approach to testing enrichment of MGEEs around the ancestral chromosomal breakpoints on each of the four chromosomes with macro-rearrangements, finding that both Chr I and XXI showed significant increases in micro-rearrangement near their ancestral breakpoints, but Chr IV and VII did not (supplementary table S3, Supplementary Material online). LSGs and duplications were not enriched near any ancestral breakpoints (supplementary table S3, Supplementary Material online). Most of the genomic islands that are significantly enriched for micro-rearrangements are not near the ancestral breakpoints on the focal chromosomes (fig. 3, with the exception of two islands on Chr XXI), and none of the islands on Chr I contributed to the significance of the genome-wide patterns (fig. 4). Enrichment driven by a higher rate of MGEEs near ancestral breakpoints, therefore, does not seem to be a general explanation for the patterns we found but might explain the enrichment found in the two islands that overlap the breakpoints on Chr XXI.

### MGEEs are Enriched for Marine Versus Freshwater Differential Expression

**Fig. 5.**
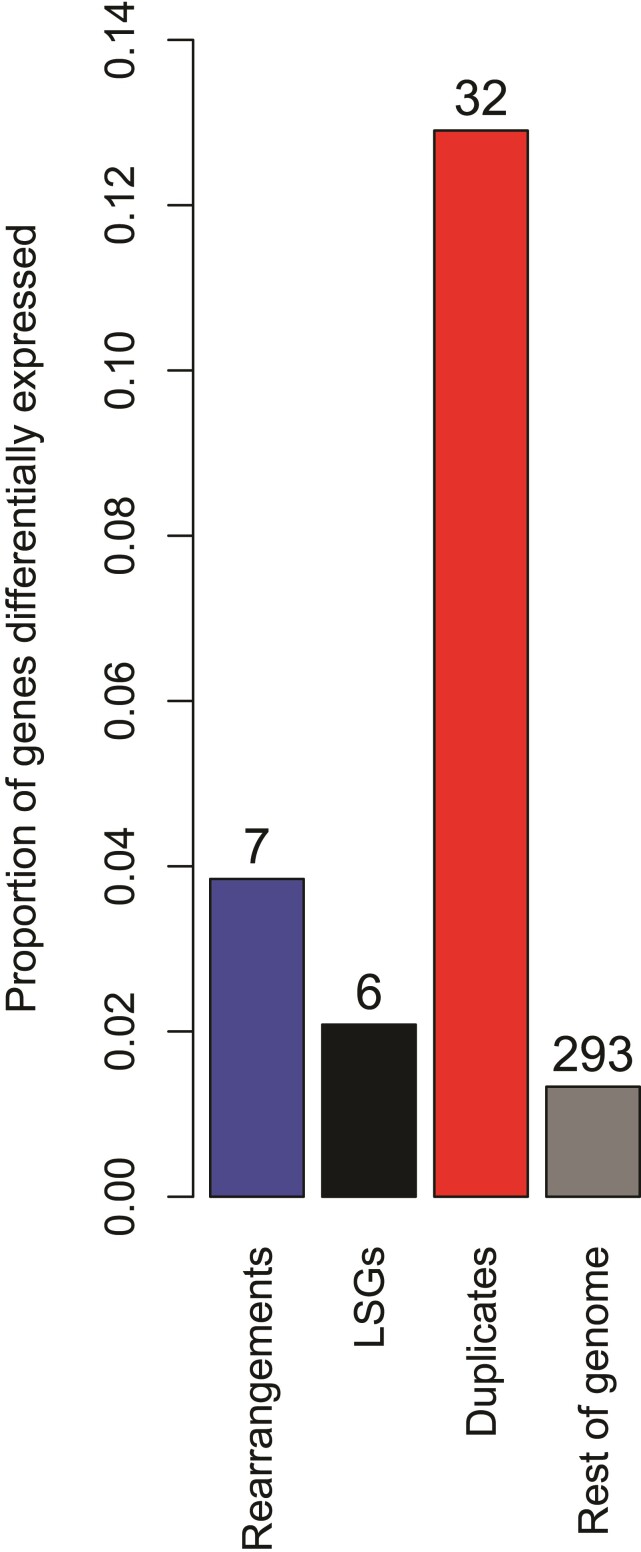
Proportion of genes involved in the three types of MGEE that are differentially expressed among freshwater versus saltwater threespine stickleback ecotypes. Numbers above each bar indicate the number of genes; for duplicates, all genes in group of related duplicates are counted as a single gene.

To examine whether the MGEEs tend to involve genes that are functionally important, we used a recently published dataset on differential gene expression among freshwater and marine stickleback ecotypes raised in a common environment, assayed in gill tissue (Verta and Jones 2019). Out of the 21,855 genes in our high confidence set that had not experienced an MGEE, 293 were identified as being differentially expressed between these ecotypes (1.3%; fig. 5). We found significantly higher rates of differential expression in genes involved in micro-rearrangements (3.8%; binomial test *P* = 0.02) and duplications (12.9%; *P* < 10^−19^) but not LSGs (2.1%; *P* = 0.26). As there were very few of these genes overall, it was not possible to test enrichment within chromosomes, however, some intriguing patterns are apparent. Of the seven differentially expressed genes on ChrIV that were involved in an MGEE, six of them cluster within the significant regions near *Eda*/*Msx2a* identified in fig. 3 (three genes involved in groups of duplications and three LSGs). Similarly, all five of the MGEEs on ChrVII that were also differentially expressed are found within the first 3.5 Mbp of the chromosome, where there is a significant enrichment of duplications within a PNW genomic island. By contrast, differential expression was not found in any of the genes involved in the MGEEs within the genomic islands on ChrXXI near *Tfap2a* and *Bmp6*.

### Chromosomal Distribution of Transposable Elements

**Fig. 6.**
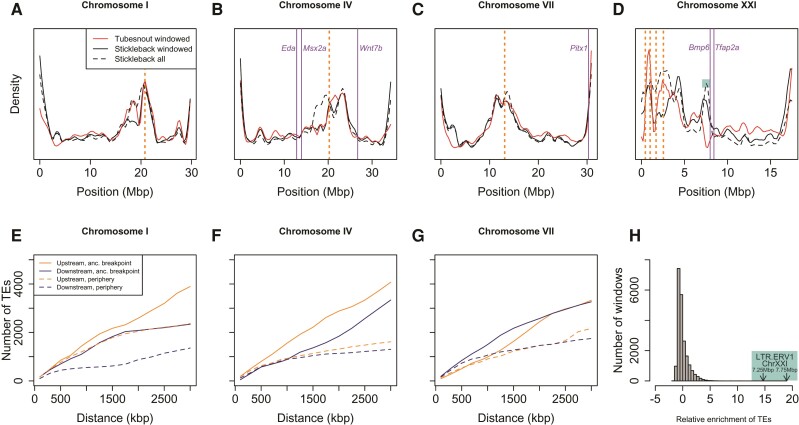
Distributions of TEs in threespine stickleback and tubesnout in the four chromosomes with macro-rearrangements. Panels (*A–D*) show the density of TEs from the windowed-ortholog analysis in both species, as well as the raw density of all TEs in threespine stickleback, which includes those that do not fall within the windows around identified orthologs. Panels (*E–G*) show the number of TEs in threespine stickleback within a given distance upstream and downstream from the ancestral breakpoint of the macro-rearrangement, and compare this to the number of TEs found in the same distance from either end of the contemporary periphery of each chromosome. Panel (*H*) shows the relative abundance of different classes of TE within 500 kb windows, with the highest enrichment shown for LTR.ERV1 elements in two windows on ChrXXI, highlighted in the colored box, with the corresponding region highlighted in panel (*D*).

Transposable elements (TEs) can drive genome evolution, either by directly moving genes during transposition or by facilitating rearrangements through unequal recombination (Petes and Hill 1988; Mani and Chinnaiyan 2010; Lisch 2013). Across the whole genome, the numbers of micro-rearrangements and duplications correlated strongly with the density of TEs when calculated on 500 kbp moving windows (Kendall's *τ* = 0.21, *P* < 10^−14^ and *τ* = 0.11, *P* < 10^−4^, respectively), with patterns of elevated TE density in the peripheral chromosomal regions (supplementary fig. S7*D*, Supplementary Material online). TEs were also significantly enriched near the ancestral breakpoints of the macro-rearrangements on chromosomes I, IV, and VII (fig. 6*A–G*), even after correcting for the increased density expected if the macro-rearrangements had not occurred and these had remained as peripheral regions (supplementary table S5, Supplementary Material online). By contrast, LSGs did not correlate with TE density (*τ* = 0.03, *P* = 0.25).

To compare patterns of TE occurrence in threespine stickleback versus tubesnout, we used a 500 kb moving-window analysis in threespine stickleback, identifying all genes within each window with syntenic and collinear mappings in tubesnout. For each of these genes, we included all TEs mapped within 50 kb, and then counted all unique TEs within each 500 kb window (see Materials and Methods for details). We found similar patterns of overall TE density in both species, with small localized increases or decreases in density found in many homologous chromosome regions in both species (fig. 6*A–D*). The most striking departure from this similarity is observed just upstream from the region of *Bmp6* and *Tfap2a* on ChrXXI that harbors significant enrichment of all three types of MGEE, where there is dramatic enrichment of LTR.ERV1 elements found only in threespine stickleback (fig. 6*D*). Two 500 kbp windows centered around 7.25 and 7.75 Mbp on ChrXXI show the greatest enrichment observed for any common type of TE anywhere in the genome, with 162 and 208 LTR.ERV1 elements, respectively, which is >14 standard deviations above the mean of 4.9 per window (fig. 6*H*).

## Discussion

Our aim was to test whether macro- and micro-rearrangements have affected the genome architecture of loci that contribute to local adaptation. The evidence on this question is mixed: we found no MGEE “smoking guns” directly involving known candidate genes for local adaptation, which were all found as single copies in syntenic locations in all species. However, we did find many patterns that are consistent with local adaptation causing evolution in genome architecture, with significant enrichment of micro-rearrangements and duplications around genomic islands (fig. 4), pronounced enrichment of all three types of MGEE around genomic islands on the focal chromosomes (fig. 3), increased involvement of differentially expressed genes in duplications and micro-rearrangements (fig. 5), and macro-rearrangements in all three focal chromosomes (fig. 2).

On the macro-scale, we identified a previously unknown pattern of complex rearrangements in the first 2.5 Mbp on ChrXXI, which shows that all three focal chromosomes commonly involved in local adaptation have undergone either fusions or complex rearrangements, which is unlikely to have occurred at random (*P* ≤ 0.003). This is particularly noteworthy given that in both threespine and ninespine stickleback, chromosome IV has likely been created by fusions of the same ancestral chromosomes, which would be very unlikely to happen by chance. These patterns are consistent with another analysis of macro-rearrangements using a de novo assembly of the fourspine stickleback (Liu, Chen, et al. 2021; Liu, Roesti, et al. 2022), and strongly suggest an adaptive mechanism driving macro-rearrangements in stickleback. Finding macro-rearrangements associated with local adaptation is consistent with population genetic predictions: if two locally adapted loci experience selection of *s_a_* and *s_b_* and are separated by recombination at rate *r*, then they will experience an advantage due to linkage whenever *r* < *s_a_s_b_*/*m*,  where *m* is the migration rate (Yeaman et al. 2016). Given that *Eda* experiences particularly strong selection, with estimates of *s* ∼ 0.5 (Schluter et al. 2021), an advantage for linkage with *Eda* would extend to other locally adapted alleles (with *s* ∼ *m*) across the length of ChrIV (i.e., at distances up to *r* = 0.5). As such, selection acting on *Eda* and any locally adapted alleles on the other pre-fusion chromosome could have yielded a benefit of linkage strong enough to drive the fixation of these ancestral fusions (as per Guerrero and Kirkpatrick 2014).

On the micro-scale, we found significant patterns of enrichment of rearrangements and duplications within genomic islands of local adaptation (fig. 4), with particular enrichment of all three types of MGEE near *Tfap2a* and *Bmp6* and enrichment of duplications and LSGs in genomic islands in the region of *Eda* and *Msx2a* (fig. 3), although the significance of this latter pattern was more pronounced under the “single-adj” and gene density models (supplementary figs. S11 and S13, Supplementary Material online). It is unlikely that such enrichment would happen under a null model of MGEEs being driven only by mutation rate, drift, and purifying selection. Similarly, the genes involved in small rearrangements or duplications were, respectively, 2.9 and 9.9 times more likely to be differentially expressed between marine and freshwater ecotypes than non-MGEEs (fig. 5), which is very unlikely to happen if such events occur at random. This could potentially be explained if purifying selection to eliminate new MGEEs is weaker when they involve genes with evolutionarily labile expression—the observed enrichment could then be driven by a lack of MGEEs involving genes with conserved patterns of gene expression. Finally, we found that genes involved in duplications tended to be enriched for GO terms related to olfactory receptor activity and hemoglobin gas transport (supplementary table S4, Supplementary Material online), both of which may be important for local adaptation to marine versus freshwater. Duplication of genes involved in olfaction is common among vertebrates (Niimura et al. 2014; Vandewege et al. 2016) and recent evidence has found increases in copy number of certain subfamilies of olfactory receptors in freshwater fish species, relative to marine ones (Liu, Chen, et al. 2021; Liu, Roesti, et al. 2022). Similarly, duplication of globin genes allows for the synthesis of different hemoglobin forms (Storz 2016), and in fish the evolution of pH-specific globin isoforms is thought to help them colonize a wide variety of aquatic environments (Randall et al. 2014; Storz 2016). For example, in red drum (*Sciaenops ocellatus*), there is evidence for changes in the expression of hemoglobin isoforms during acclimatation to hypoxia, supporting the idea that an increased repertoire of hemoglobin genes can help species deal with environmental challenges (Pan et al. 2017). Taken together, many MGEEs have evidence consistent with a role in local adaptation to freshwater versus marine environments, but functional characterization of the genes involved is needed to more concretely establish this.

The large number of putative LSGs identified here suggests that de novo gene birth might be important in stickleback (particularly near *Tfap2a* and *Bmp6*), but our confidence in these results is limited by difficulties involved with the correct identification of LSGs. A recent analysis showed how even under a model of uniform evolutionary rate, homology-based search approaches could fail to detect true orthologs and therefore often misidentify a shared gene as an LSG (Weisman et al. 2020). On the other hand, there are some well-substantiated examples of de novo gene birth (Schlötterer 2015; Van Oss and Carvunis 2019) and when this happens, presumably the LSG would still share some nucleotide homology with other closely related species (in the region where the gene was “born”), but without the expression and function that are hallmarks of a “real” gene. To allow for this latter possibility, we conducted our enrichment analyses with a permissive filtering criterion to allow for partial homology. However, we caution that many of these putative LSGs require further validation by studying function and expression more deeply, and consider the “stringent” list of genes included in the archived data as the higher confidence set of putative LSGs.

It seems likely that TEs are at least partly responsible for these patterns in MGEEs, whether through promoting higher rates of rearrangement through unequal recombination (Petes and Hill 1988; Mani and Chinnaiyan 2010), or more directly through transposon-mediated movement (Lisch 2013). The region just upstream of *Tfap2a* and *Bmp6* on ChrXXI harbors the greatest enrichment of any TE anywhere in the genome, with 33–42× the average number of LTR.ERV1 elements (fig. 6*H*). While it is possible that these patterns are the neutral result of rearrangement rate, this would not explain why such a concentration happens to occur adjacent to these two candidate genes which also harbor significant enrichment of MGEEs in genomic islands.

Taken together, these results and those of another comparative study using fourspine stickleback (Liu, Chen, et al. 2021; Liu, Roesti, et al. 2022) are consistent with the patterns expected if local adaptation drives genome evolution, but are not conclusive, given the retrospective nature of the analysis. Functional analysis of the genes involved in MGEEs would help strengthen the evidence for local adaptation as a driving force shaping these rearrangements. Further studies on other species that experience strong and persistent divergent selection and local adaptation over millions of years would help establish whether this pattern is the result of common process or is particular to the stickleback clade. Given that rates of rearrangement are particularly high in plants (Zhao and Schranz 2019), it seems possible that local adaptation in plants would more readily result in this kind of evolution in genome architecture.

## Materials and Methods

### De Novo Assembly of the Tubesnout Genome

A single male tubesnout specimen supplied by Living Elements (Vancouver BC) was used for all genome sequencing and assembly-related experiments in this study. The genome assembly was performed by GCEv1.0. with 18.5 Gb (∼40×) error-corrected Pacbio reads. High molecular weight genomic DNA was isolated and purified with the QIAGEN Genomic-Tip from muscle tissue stored at −80 °C. One 20 kb insert-size SMRTbell library was constructed with the Pacbio P6 v2 binding Kit, and was sequenced on 53 SMRTcells. SMRTanalysis V4.0 was used for processing and filtering the raw reads to get reads-of-insert (ROI). The ROIs longer than 3.5 kb were chosen as seed reads to generate error-corrected consensus sequences with higher accuracy for genome assembly.

We employed the diploid aware “FALCON + FALCON-unzip” approach to assemble the phased haploid genome sequences of tubesnout (Chin et al. 2016; see Dryad archive for config files). FALCON v0.5 was first used to produce the sets of primary/associated contigs representing the divergent allelic variants. All the contigs were then conveyed to the FALCON-unzip module, during which the phased haplotigs were separated based on the information of heterozygous SNPs identified by mapping the ROIs to the FALCON primary/associated contigs.

The Proximo Hi-C library with the insert size of the sheared ligations of ∼600 bp was constructed from 95% ethanol preserved muscle tissue by Phase Genomics, and was sequenced on the Illumina Hiseq 4000 platform, generating 113,119,916 paired-end reads with 100 bp read-length. The Hi-C scaffolding was performed with the 3D de novo assembly (3D DNA) pipeline. Firstly, the Hi-C reads were mapped to the draft-assembled contigs with Juicer to generate the Hi-C contact matrix. Then we ran the 3d-DNA analysis to create an interactive heatmap, which was manually revised for the few remaining errors like haplotigs residual and incorrect placement of the contigs. The final 23 chromosome-scale super scaffolds were exported with the run-asm-pipeline-postreview.sh script. The contigs that could not be assigned to any supper scaffolds were concatenated into chromosome UN with 500 Ns separating each contig.

### Gene Identification and De Novo TE Annotation

The threespine stickleback genome (Peichel et al. 2017) and tubesnout genome were first soft-masked for repeats using Repeatmasker with Repbase and custom libraries created by Repeatmodeler (v1.0.11; http://www.repeatmasker.org/RepeatModeler). For gene structure annotation of the threespine stickleback genome, we followed the Braker2 pipeline (Brůna et al. 2020) using online RNA-seq data from different tissues (SRR5237998, SRR5420700, SRR4116640, SRR1390640, SRR1390630, SRR5420689) and the protein sequences from the existing Ensembl annotation (ftp://ftp.ensembl.org/pub/release-90/fasta/gasterosteus_aculeatus/) to train the gene prediction tools GeneMark-ET (Lomsadze et al. 2014) and AUGUSTUS (Stanke et al. 2006). For the tubesnout genome, muscle RNA-seq data and the threespine stickleback protein sequences identified from the prior reannotation were used in Braker for this novel genome. In threespine stickleback, the 25,439 identified genes were validated by either presence in the Broad annotation, or >0.1 TPM RNA-seq reads from tissues of brain, liver, gill, kidney, head kidney, spleen, muscle, skin, eye, heart, and testis tissues, or the result of target restricted assembler, aTRAM (Allen et al. 2018) with the same RNA-seq dataset. We used an automated software package (EDTA; Ou et al. 2019) for de novo genome-scale TE annotation in threespine stickleback. We then assessed if any of the above 25,439 genes were likely mis-annotated TEs using two methods. First, we used protein BLAST+ (Camacho et al. 2009) to map gene sequences against the TE library generated from EDTA, and removed any gene with >50% of its sequence having hits to TEs with >75% nucleotide identity. Second, we assessed the overlap between TE annotations and gene annotations and removed any gene with >10% of its exon sequence overlapping with TE annotations. We used these cutoffs as the default approach to curate a final annotation, yielding 23,185 high confidence genes, which are used for all downstream analyses unless specifically noted.

### Identifying Macro-Rearrangements by Ancestral Genome Reconstruction (Method 1)

To reconstruct macro-rearrangements, we conducted rigourous identification of orthologs with 10 fish species (including tubesnout), using OMA standalone v2.2.0 (Altenhoff et al. 2015), coupled with genome reconstruction of the threespine stickleback—tubesnout ancestor using ANGES v1.01 (Jones, Grabherr, et al. 2012; Jones, Rajaraman, et al. 2012) (see supplementary methods, Supplementary Material online), and identified 19,563 orthologs with high confidence. Genome maps for these species plus threespine stickleback and tubesnout were prepared based on gene position information extracted from the gff3 or gtf files. Gene start and end positions were calculated as the average of CDS midpoints ± 1 base pair to avoid the occurrence of overlapping gene positions, which are not supported by the genome reconstruction software ANGES v1.01 (Jones, Grabherr, et al. 2012; Jones, Rajaraman, et al. 2012). A few remaining overlaps between gene positions were resolved manually to obtain an unambiguous order of genes for each genome. ANGES input files were generated from these genome maps and from the best-scoring phylogenetic tree computed with a set of 2,504 common one-to-one orthologs. The genome of the *G. aculeatus*—*A. flavidus* ancestor was reconstructed using the ANGES master pipeline (anges_CAR.py) and options markers_doubled 1 (infer ancestral marker orientation), markers_unique 2 (no duplicated markers), markers_universal 1 (no missing markers in ingroup), c1p_telomeres 0 (no telomeres), and c1p_heuristic 1 (using a greedy heuristic), including as outgroup all nine additional species. A total of 46,363 *Ancestral Contiguous Sets* (ACS; Jones, Grabherr, et al. 2012; Jones, Rajaraman, et al. 2012) were identified by ANGES, of which 42,993 ACS were organized into 249 CARs (3,370, or 7.3%, of ACS were discarded by the program). These CARs comprised a total of 14,461 ancestral markers, and 12,474 of them (86.3%) were grouped into the 23 largest CARs (i.e., major ancestral chromosomes). Putative fusions between ancestral chromosomes were identified by visually inspecting assignments of CARs to threespine stickleback and tubesnout chromosomes (fig. 2) using the R package rearrvisr (Lindtke and Yeaman 2020).

### Identifying Macro-Rearrangements and MGEEs by Homolog Mapping (Method 2)

To reconstruct the history of macro-rearrangements and MGEEs in the threespine stickleback lineage, we first used gmap (Wu and Watanabe 2005) to map all 23,185 putative genes from threespine stickleback to identify their closest homologs in *P. sinensis* (21,885 mappings), tubesnout (20,995 mappings), and seabass (18,989 mappings) genomes (with >100 bp of sequence matching at >75% ID). Because the tubesnout and seabass genomes are assembled to near chromosome scale, we used these for our main analysis, and used the *P. sinensis* genome, which is somewhat more fragmented, to aid in resolving uncertain synteny relationships. Macro-rearrangements were identified by visual inspection of chromosomal synteny plots (supplementary figs. S4–S6, Supplementary Material online). For micro-rearrangements, three types of noncorrespondence in the spatial organization of these homologs were identified at the gene-level: (A) LSGs unique to sticklebacks (i.e., present in *P. sinensis* and threespine stickleback but absent from tubesnout and seabass); (B) genes where the homolog is present on a nonsyntenic chromosome in at least one species, which we call putative rearrangements; (C) cases where multiple genes on a single chromosome in threespine stickleback map to a single homolog in tubesnout, which we refer to as duplications.

For the LSGs (case A), we failed to identify any match for 1,340 of the 23,185 high confidence stickleback genes in either tubesnout or seabass, 608 of which could also be successfully mapped to the *P. sinensis* genome. There are four plausible explanations for their occurrence: (1) they are bioinformatic errors and not real genes, (2) they have a true homolog in another species but evolved rapidly in their sequence, thereby obscuring orthology relationships, (3) they are genes that evolved through de novo “gene birth” in stickleback, or (4) homologous genes were lost independently in both the tubesnout and seabass lineages. We assume that (4) is unlikely to have occurred commonly, so we do not further consider it here. To attempt to identify stickleback LSGs and rule out the first two explanations, we conducted additional filtering, removing any genes with a successful BLAST+ (Camacho et al. 2009) hit to the NR boneyfish database using a permissive threshold (*e* < 0.001), leaving 299 genes that appear to be unique to clade including *Gasterosteus* and *Pungitius* stickleback. To further check whether the above steps failed to detect a true homolog in tubesnout, we identified the homologous genomic region between tubesnout orthologs of the genes flanking the putative LSG in threespine stickleback. We then conducted a BLAST+ search of the putative LSG against this restricted region (*e* < 0.001) and excluded any cases with >90% coverage (“permissive” filter; keeping 288 genes) or any cases with a hit at any level of coverage (“stringent” filter; keeping 70 genes). For all enrichment testing below, we report results based on the permissively filtered set of 288 genes.

For among-chromosome mismatches (case B), these could arise from true rearrangements, genome mis-assembly in one or more species, or as bioinformatic errors in ortholog identification, but we refer to them as rearrangements for simplicity. For each threespine stickleback chromosome, the homologous chromosome(s) were identified in tubesnout and individual genes in threespine stickleback were considered as putative micro-rearrangements if none of the best-hit mappings from gmap were in a syntenic location (we considered up to five mappings for each gene). This included both genes that had a one-to-one mapping relationship and genes where multiple stickleback genes on different chromosomes mapped to the same location in tubesnout (many-to-one). Several steps were then taken to exclude cases that more likely arose from bioinformatic errors and to attempt to infer where in the phylogeny the rearrangement may have occurred, excluding cases that could confidently be ascribed to have occurred in the tubesnout lineage (see supplementary methods, Supplementary Material online).

For both LSGs (A) and putative rearrangements (B), we conducted a follow-up filter using BLAST+ (tblastx) to attempt to map each putative LSG or rearranged gene to the homologous area of the tubsnout genome, spanning the region between the closest syntenic neighboring orthologs upstream and downstream of the focal gene (as per Weisman et al. 2020). For any case with a BLAST+ hit of *e* < 0.001 within this restricted region, the putative LSG/rearranged gene was excluded from further analysis. When flanking orthologs could not be identified readily, as would occur in areas with complex macro-rearrangements (i.e., ChrXXI), this final test was not conducted.

For case (C), we identified duplications as cases where at least two genes on the same chromosome in stickleback have their highest mappings to a single-copy gene in tubesnout and are also single-copy in seabass. Putative duplications were removed if they did not also present as a duplication when the same analysis was repeated comparing stickleback to seabass, as this would be more parsimoniously explained by a deletion in tubesnout.

### Testing Enrichment of MGEEs and TEs

To assess whether MGEEs or TEs were enriched near particular regions of the genome, for each type of event, we counted all occurrences within <*x* bp upstream and downstream of the region of interest, and allowed *x* to vary from 100 kb to 3 Mbp (seven increments), in order to examine clustering at different scales. For each *x*, we constructed a null distribution by randomly redrawing the chromosome based on the number of genes, randomly redrawing the start positions of all events of the same type (according to one of four density distributions) and recording how many events fall within the same increment, using 10,000 replicates. The empirical *P*-value was calculated as the proportion of null distribution replicates that equaled or exceeded the observation. Where a rearrangement event included more than one gene, this was counted as a single event; for a duplication event, any adjacent copies separated by <1 Mbp were counted as a single event to discount a signal of clustering caused by multiple tandem duplicates of the same gene.

As we observed that the distribution of both MGEEs and TEs tended to be nonuniform across the chromosome (supplementary fig. S7, Supplementary Material online), we repeated the above approach under four models specifying the probability of event occurrence based on the relative position along the chromosome. First, we visually assessed whether there were differences between the spatial distribution of MGEEs among-chromosome morphologies (i.e., acrocentric, metacentric, telo-centric, as per Urton et al. (2011). As there were no striking differences between these types (supplementary fig. S8, Supplementary Material online), we opted to treat all types of chromosomes equally, given uncertainties in centromere position and how to rescale the relative position in such cases. We constructed probability density models for each type of event based on observations from all chromosomes that had not undergone macro-rearrangements and folded each chromosome in half, such that the relative positions scale from 0 to 0.5 (“reflected rescaling”). For each type of event we fit a bounded density model to the reflected rescaled data using the bde library in R (v1.01, with “boundarykernel” and *b* = 0.15), which we termed the “single-adj” density model. Given that chromosomes I, IV, and VII experienced simple fusions or translocations (rather than the complex rearrangements found in ChrXXI), for these chromosomes we also fit the above bde model to each side of the breakpoint of the macro-rearrangement individually, scaled to the length of each segment (which we term the “double-adj” model; see supplementary fig. S9, Supplementary Material online). The parameters for density models were determined based on subjective visual assessment of the goodness of fit, before running the enrichment tests and no further alteration of these parameters was made, to avoid *P*-hacking. In the main body of the manuscript, we use the “double-adj” model for chromosomes I, IV, and VII (as these chromosomes are fusion products) and the “single-adj” model for chromosome XXI (as it has only a small region of complex rearrangement at one end). We report results for the single-adj model, a uniform distribution (“flat” model), and a model scaling MGEE occurrence by gene density in the supplementary materials, Supplementary Material online.

### Differential Expression

We were interested in assessing overlap between our rearrangements, LSGs, and duplications with the genes identified as “parallel diverged” in their expression between marine versus freshwater ecotypes by Verta and Jones (2019). As their analysis used the BROAD S1 genome, it was necessary to map the nucleotide sequences for these genes to the Peichel et al. (2017) annotations used here, which was done using BLAST against the cDNA. These mappings were sorted by *z*-score and *e*-value and the best match was determined based on highest sequence overlap. Analysis of enrichment for duplicated genes treated all copies of a duplicate as a single gene, which was counted as differentially expressed if at least one of the duplicates had a best-hit mapping from the Verta and Jones candidates.

### TE Density

To compare the chromosomal landscapes of TE density between threespine stickleback and tubesnout, we used the simple gene mappings from method 2 that were not involved in any MGEE and were thus collinear and syntenic between the two species. We conducted our analysis in 500 kb windows; within each window we identified all TEs that fell within 50 kb upstream or downstream of each collinear and syntenic gene in each species, and then counted the number of unique TEs within each 500 kb window. To study the chromosomal distribution of each type of TE in threespine stickleback, we excluded any TEs with a mean density of <1 copy per 500 kb window, and then converted their relative density within each window to a *z*-score based on the mean and standard deviation of occurrences per window across the whole genome.

## Supplementary Material

Supplementary data are available at *Genome Biology and Evolution* online (http://www.gbe.oxfordjournals.org/).

## Supplementary Material

evac075_Supplementary_Data

## Data Availability

The genomic resources, data, and scripts needed to conduct the main analyses in this paper are included in the Dryad repository (https://doi.org/10.5061/dryad.1c59zw3w3).
