# Supplementary material for: Local Adaptation and the Evolution of Genome Architecture in Threespine Stickleback
Source: Genome Biol Evol. 2022 May 20;14(6):evac075. doi: 10.1093/gbe/evac075 (PMC9178229; doi:10.1093/gbe/evac075)
Supplement: evac075_Supplementary_Data [file evac075_supplementary_data.pdf]

Local adaptation and the evolution of genome architecture in threespine stickleback

Supplementary materials

METHODS

Notes on the historical development of these methods
This study was begun in 2015 to test the prediction of Yeaman (2013) that rearrangements might be involved in the evolution of genomic islands. Initial consultations with experts attending the triennial stickleback meeting in 2015 identified Chr IV, VII, and XXI as the regions of the genome most important in driving local adaptation, based genome scans of marine-freshwater divergence (Hohenlohe *et al*. 2010; Jones *et al*. 2012), a comprehensive QTL mapping experiment focusing on traits commonly involved in adaptative divergence between marine and freshwater ecotypes (Miller et al. 2014), and well-substantiated candidate genes driving phenotypes that contribute to marine-freshwater divergence: *Eda* (ChrIV; Colosimo *et al*. 2005), *Pitx1* (ChrVII; Shapiro *et al*. 2004), and *Bmp6* (ChrXXI; Cleves *et al*. 2014). Initial attempts were made at delineating boundaries of genomic islands, but it was clear that results would be highly sensitive to such arbitrary choices and we were concerned about engaging in *p*-hacking if we revised methods and re-tested multiple times. New meta-analyses were then published combining data from stickleback genome scans (Samuk et al. 2017) and QTL studies (Peichel and Marques 2017), further complicating the issue about how to delineate the boundaries of genomic islands. These studies still pointed to the focal chromosomes being most important for local adaptation (Table S1), but we decided to shift to an approach to test enrichment centered around candidate genes instead, as this would have few arbitrary choices about boundaries. This method was implemented and eventually submitted for publication. Reviewers recommended a more standardized approach to enrichment testing using boundaries of genomic islands recently identified by Kingman et al. (2021), and so we applied our previous method to this new dataset, retaining the same size of 3Mbp windows around each genomic island. Results for the earlier method testing enrichment around candidate genes are shown in table S4.

Data preparation for identifying rearrangements by ancestral reconstruction (method 1):
In addition to the annotated genomes of threespine stickleback (Peichel et al. 2017) and tubesnout described in the main manuscript, peptide sequences and annotation information (fasta and gff3 or gtf files) for genes from nine fish species were downloaded from the following publicly available resources: zebrafish, *Danio rerio* GRCz11, Ensembl release 93, jul2018.archive.ensembl.org/Danio_rerio; European sea bass, *Dicentrarchus labrax* v1 (Tine et al. 2014): seabass.mpipz.mpg.de/DOWNLOADS; large yellow croaker, *Larimichthys crocea* 1.0: GenBank assembly accession GCF_000972845.1; Asian sea bass (Barramundi), *Lates calcarifer* v2 (Vij et al. 2016); Nile tilapia, *Oreochromis niloticus* 2.0: GenBank assembly accession GCF_001858045.2; Japanese medaka, *Oryzias latipes* v1, GenBank assembly accession GCF_002234675.1; Japanese pufferfish, *Takifugu rubripes* FUGU4: Ensembl release 90,

aug2017.archive.ensembl.org/Takifugu_rubripes; spotted green pufferfish, *Tetraodon nigroviridis* 8.0: Ensembl release 93, jul2018.archive.ensembl.org/Tetraodon_nigroviridis; southern platyfish, *Xiphophorus maculatus* 5.0: GenBank assembly accession GCF_002775205.1.

Sequences that were shorter than 100 amino acids or contained premature stop codons were excluded. 34,572 orthologous groups were identified with OMA standalone v2.2.0 (Altenhoff et al. 2015), using the phylogeny published in (Vij et al. 2016) as a guidance tree with tubesnout placed as sister lineage to threespine stickleback (Near et al. 2013), and default settings otherwise. A single representative splicing variant per gene was identified by OMA, and alternative splicing variants were excluded from subsequent analyses. The retained number of genes placed into orthologous groups per species were 19,563, 18,381, 19,175, 21,335, 21,886, 18,570, 24,130, 20,440, 16,924, 16,527, and 21,441 for *Gasterosteus aculeatus*, *Aulorhynchus flavidus*, *Danio rerio*, *Dicentrarchus labrax*, *Larimichthys crocea*, *Lates calcarifer*, *Oreochromis niloticus*, *Oryzias latipes*, *Takifugu rubripes*, *Tetraodon nigroviridis*, and *Xiphophorus maculatus*, respectively.

Sequences of 2,881 OMA orthologous groups that only included one-to-one orthologs present in all 11 species were extracted and individually aligned with MAFFT v7.407 (Katoh 2002), using the iterative refinement method incorporating local pairwise alignment information (--localpair --maxiterate 1000 settings) and the –leavegappyregion option. Alignments with not more than 20% missing data (2,504 orthologous groups) were concatenated (1,687,665 amino acids, with 341,234 distinct alignment patterns), and the best-scoring phylogenetic tree (Figure S15) was determined with RAxML v8.2.12 (Stamatakis 2014), using the rapid bootstrap algorithm combined with a maximum likelihood search, and specifying *D. rerio* as outgroup. The protein substitution model (JTT with empirical base frequencies) and sufficient number of bootstrap replicates (50) were automatically determined by the program (-f a -# autoMRE -m PROTGAMMAAUTO --auto-prot=ml settings).

Identifying micro-rearrangements (Method 2):

Genes were identified as putative rearrangements based on non-synteny in their mapping, as described in the main manuscript methods. Several steps were then taken to check for bioinformatic errors and further curate the list of putatively rearranged genes. For the one-to-one mappings, we ignored any cases where a reciprocal mapping of the sequence from tubesnout back to stickleback was unable to identify the source location as one of the top hits, but did not apply this additional filter for the many-to-one cases. For cases where more than one putatively rearranged gene shared similar syntenic relationships (same chromosome, separated by < 50kbp), they were grouped together into a putative multi-gene rearrangement. For each gene or gene group, the evolutionary history of the putative rearrangement was then assessed by sampling a chromosomal window including 20 unique gene positions upstream and downstream of the focal homolog in tubesnout, seabass, and Amur stickleback, to avoid double-counting cases where multiple genes in threespine stickleback mapped to the same location in another species. These windows were compared between each pair of species, and if the putatively rearranged gene or gene group resided on the same chromosome/scaffold as at least 25% of the other genes within the window, it was counted as being syntenic in that species pair (again, genes were only counted as being non-syntenic and putatively rearranged if none of the top five

gmap hits was syntenic). For each gene or gene group, the synteny relationships were then used to infer the placement of the rearrangement on the phylogeny, and any genes with similar synteny/collinearity between threespine stickleback and seabass were excluded, as the mismatch likely occurred in the tubesnout branch of the phylogeny. In some cases it could not be confidently ascertained when the rearrangement occurred because of incomplete orthology identification (e.g. no ortholog identified in seabass or nonsynteny in all contrasts), and these cases were retained as putative rearrangements. As an additional filter, we checked the mapping of putatively rearranged genes in the Glazer *et al*. (Glazer et al. 2015) stickleback genome assembly, as a total of 35 of these genes are mapped to a different chromosome than in the Peichel genome (Peichel et al. 2017). However, in none of these 35 cases was the gene's position in the Glazer assembly syntenic with the position in the tubesnout assembly, so we retained all 35 cases as putative rearrangements. As another filter to exclude errors by gmap, we ignored any putatively rearranged genes that had a significant genome-wise BLAST+ hit ($e < 10^{-10}$) within the region that would be syntenic in either tubesnout or seabass.

Gene functional annotation and GO analysis of rearrangements and duplications
To test whether genes involved in duplications and rearrangements in threespine stickleback tended to have particular biological functions, we did a GO enrichment analysis of these gene sets. First, we did the functional annotation of all the re-arranged genes and one randomly selected gene from each group of duplicated genes. We did not include the few rearranged and duplicated genes that came from the BROAD genome assembly, since those already have their functional annotations. We used the eggNOG-mapper online web resource (Huerta-Cepas et al. 2017) to do the functional annotation, using the default settings. Eggnog-mapper uses homology and phylogenetic methods to identify the best ortholog from its curated database. As expected, most of our genes were annotated with the stickleback BROAD functional annotations (see Dryad archive). We submitted these annotations to g:GOst module of the g:Profiler online web resource (Reimand et al. 2007; Raudvere et al. 2019) for functional enrichment analysis, selecting threespine stickleback as "Organism" (which makes uses the BROAD S1 threespine stickleback annotations as the background for the analysis) and all other settings were left to their default values. Genes annotated from sources other than the BROAD S1 or found to be duplicated were automatically removed from the analysis.

RESULTS

Macro-rearrangements in stickleback:
Reconstruction using method 2 showed that chromosome XXI has undergone a complex series of rearrangements within the first 2.7Mbp of one end, with regions that are homologous to four different tubesnout chromosomes over spans of at least 5 genes (Figure S6A). This architecture for ChrXXI appears fixed in stickleback species, as neither the *Pungitius* linkage map (Rastas et al. 2016) nor the *Apeltes* FISH data (Urton et al. 2011) reported inconsistencies in synteny conservation across all sticklebacks, and both threespine and *P. sinensis* show similar patterns when compared to tubesnout

(Figure S6). A number of these rearranged regions appear to have undergone a series of duplications in the stickleback lineage, as genes at different positions in stickleback were mapped to identical positions in tubesnout with gmap (Figure S6).

Reconstruction using method 1 clearly showed that threespine stickleback chromosome I evolved through a translocation of part of tubesnout chromosome I with tubesnout chromosome XXI (with the remainder of tubesnout chromosome I corresponding to threespine stickleback chromosome XIII; Figure 2). That threespine stickleback chromosomes I, IV, and VII resulted from fusions/translocations of ancestral acanthopterygian chromosomes is in line with previous work that was based on synteny with physical maps of more distantly related species (Kawase et al. 2018) although other work has characterized chromosome I as a fusion (Varadharajan et al. 2019). Both methods 1 and 2 showed that all of the other 17 chromosomes are broadly conserved in their synteny between stickleback and tubesnout (Table S1), although there are likely a number of small-scale chromosomal inversions, which could not be reliably resolved using Hi-C scaffolding.

Macro-rearrangements and MGEEs on ChrI

It is noteworthy that while not included in our list of *a priori* candidates (above), ChrI harbours *Atp1a1*, a candidate gene for local adaptation to marine vs. freshwater environments (Jones et al. 2006; McCairns and Bernatchez 2009), so the macro-rearrangement involving ChrI was perhaps also driven by positive selection. For completeness, we also studied patterns of MGEE occurrence on ChrI, and found some signal of enrichment of duplications and LSGs (Figure S12).

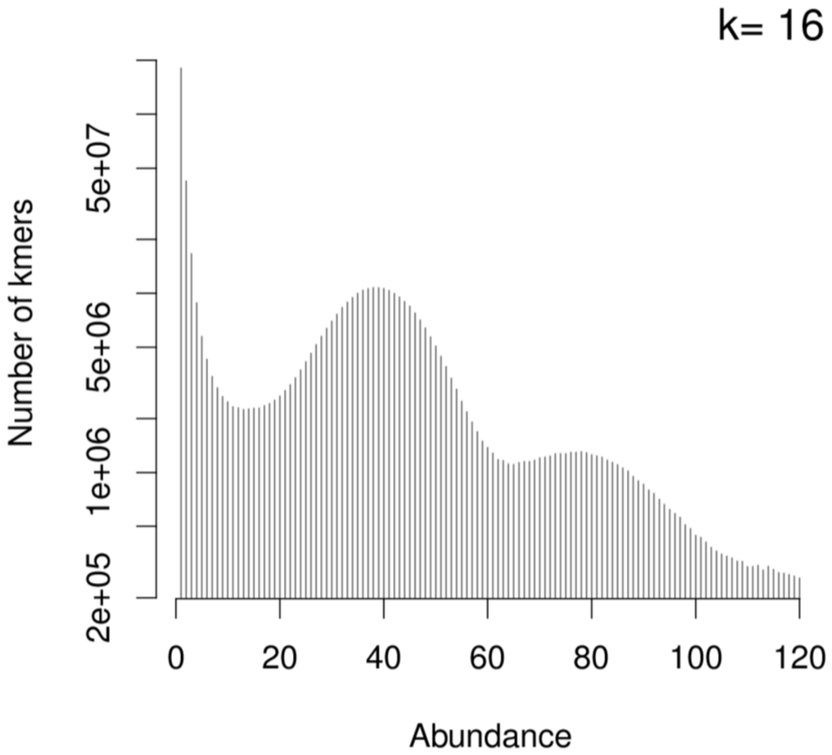

Figure S1. K-mer frequency distributions from Illumina short reads. With K=16, there is a frequency peak value at 39 which is used for haploid genome size estimation.

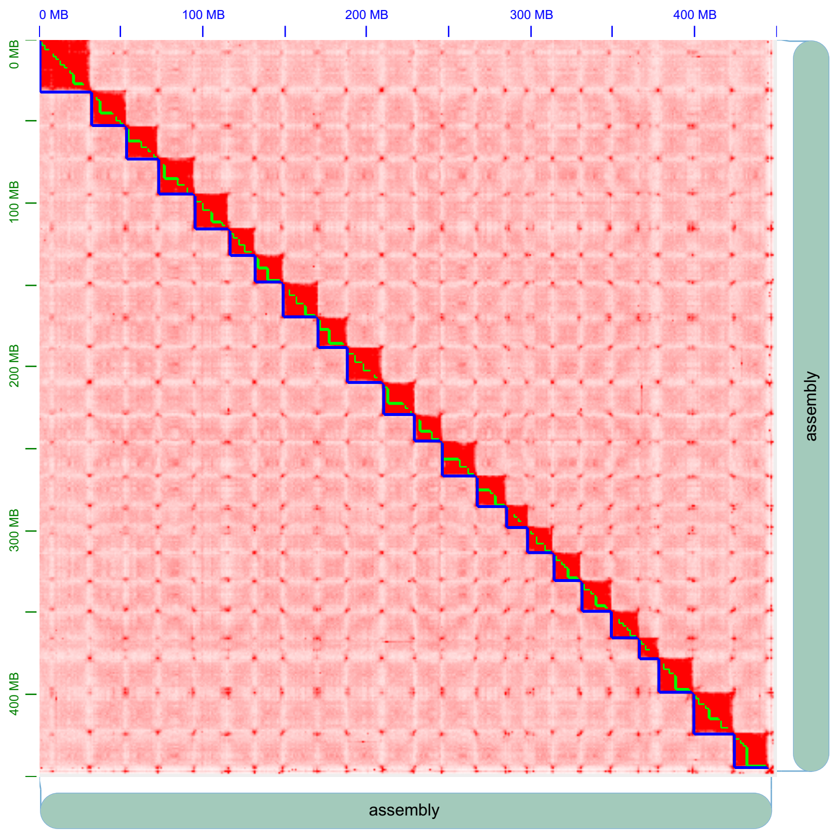

Figure S2. Post-scaffolding Hi-C contact heatmap. Contigs (boundaries shown as green squares) clustered into 23 chromosome-scale scaffolds (cScafs, boundaries shown as blue squares). The red color from light to dark indicates the increase of the Hi-C interaction density.

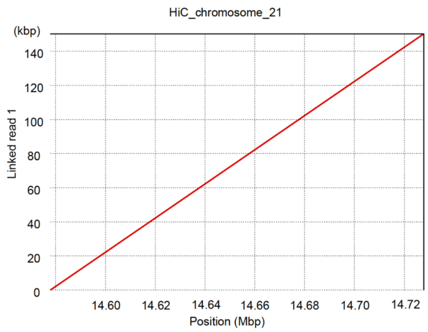
HiC_chromosome_21
(kbp)
Linked read 1
0
20
40
60
80
100
120
140
14.60
14.62
14.64
14.66
14.68
14.70
14.72
Position (Mbp)

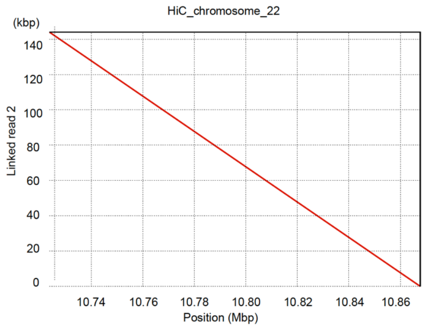
HiC_chromosome_22
(kbp)
Linked read 2
0
20
40
60
80
100
120
140
10.74
10.76
10.78
10.80
10.82
10.84
10.86
Position (Mbp)

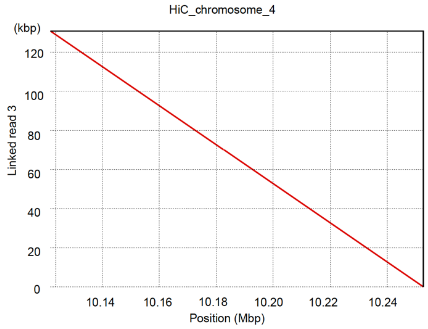
HiC_chromosome_4
(kbp)
Linked read 3
0
20
40
60
80
100
120
10.14
10.16
10.18
10.20
10.22
10.24
Position (Mbp)

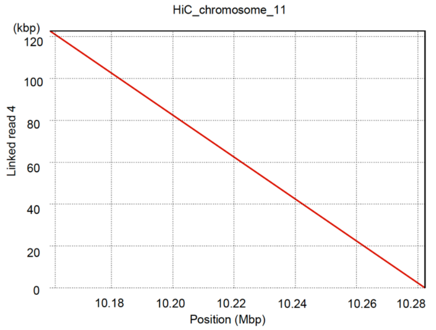
HiC_chromosome_11
(kbp)
Linked read 4
0
20
40
60
80
100
120
10.18
10.20
10.22
10.24
10.26
10.28
Position (Mbp)

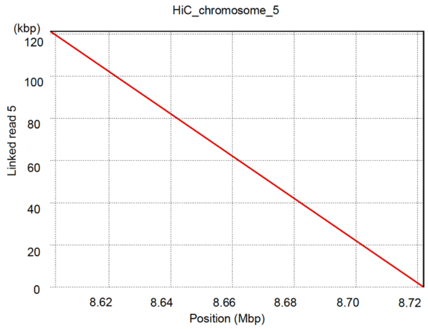
HiC_chromosome_5
(kbp)
Linked read 5
0
20
40
60
80
100
120
8.62
8.64
8.66
8.68
8.70
8.72
Position (Mbp)

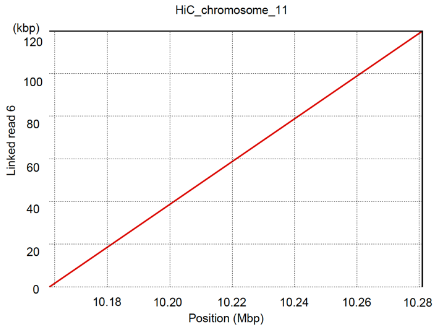
HiC_chromosome_11
(kbp)
Linked read 6
0
20
40
60
80
100
120
10.18
10.20
10.22
10.24
10.26
10.28
Position (Mbp)

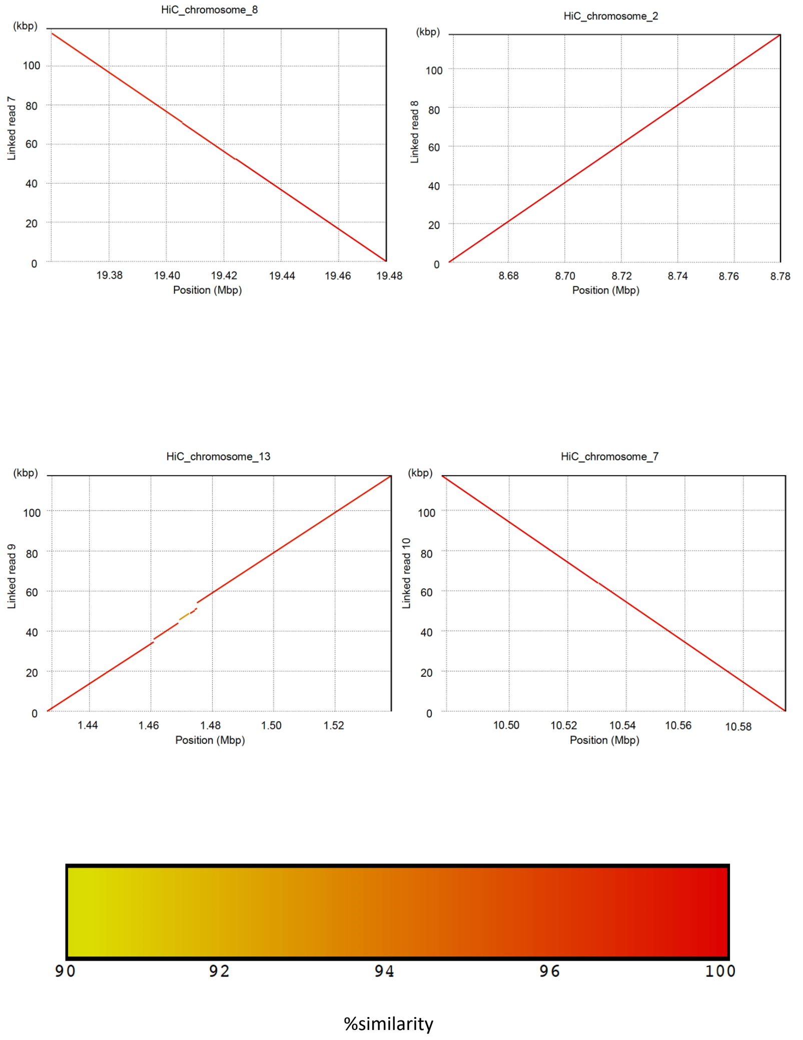

Figure S3. Alignments of linked-reads against the tubesnout genome assembly. Ten longest reads (>100kb) assembled following the orthogonal linked-reads strategy were aligned to the tubesnout chromosomal scaffolds. All linked-reads, except for the L9, show identity between 100.0% and 98.2% to the genome assembly. L9 (117,494 bp) shows slightly fragmented alignment to chromosomal scaffold 13, with the lowest identity of 93.15% at a specific region of ~3kb.

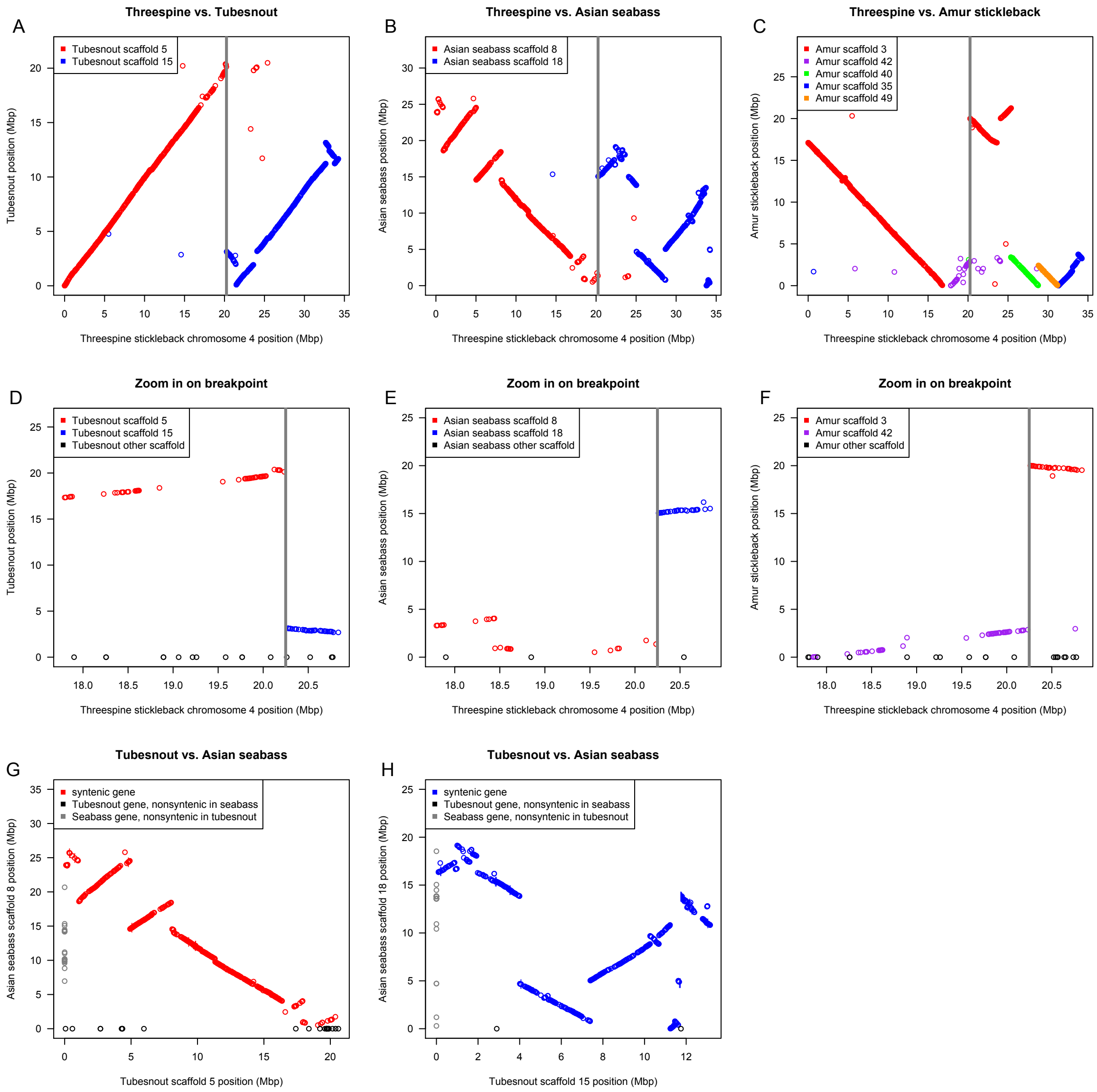

Figure S4. Patterns of synteny among scaffolds homologous to threespine stickleback chromosome IV. Each point represents the mapping of a single gene using method (2) that either supports the synteny (coloured points) or maps to some other scaffold in the assembly (black or grey; excluded from panels A-C for clarity). Vertical grey line marks the position of the mapping-breakpoint on threespine chromosome IV.

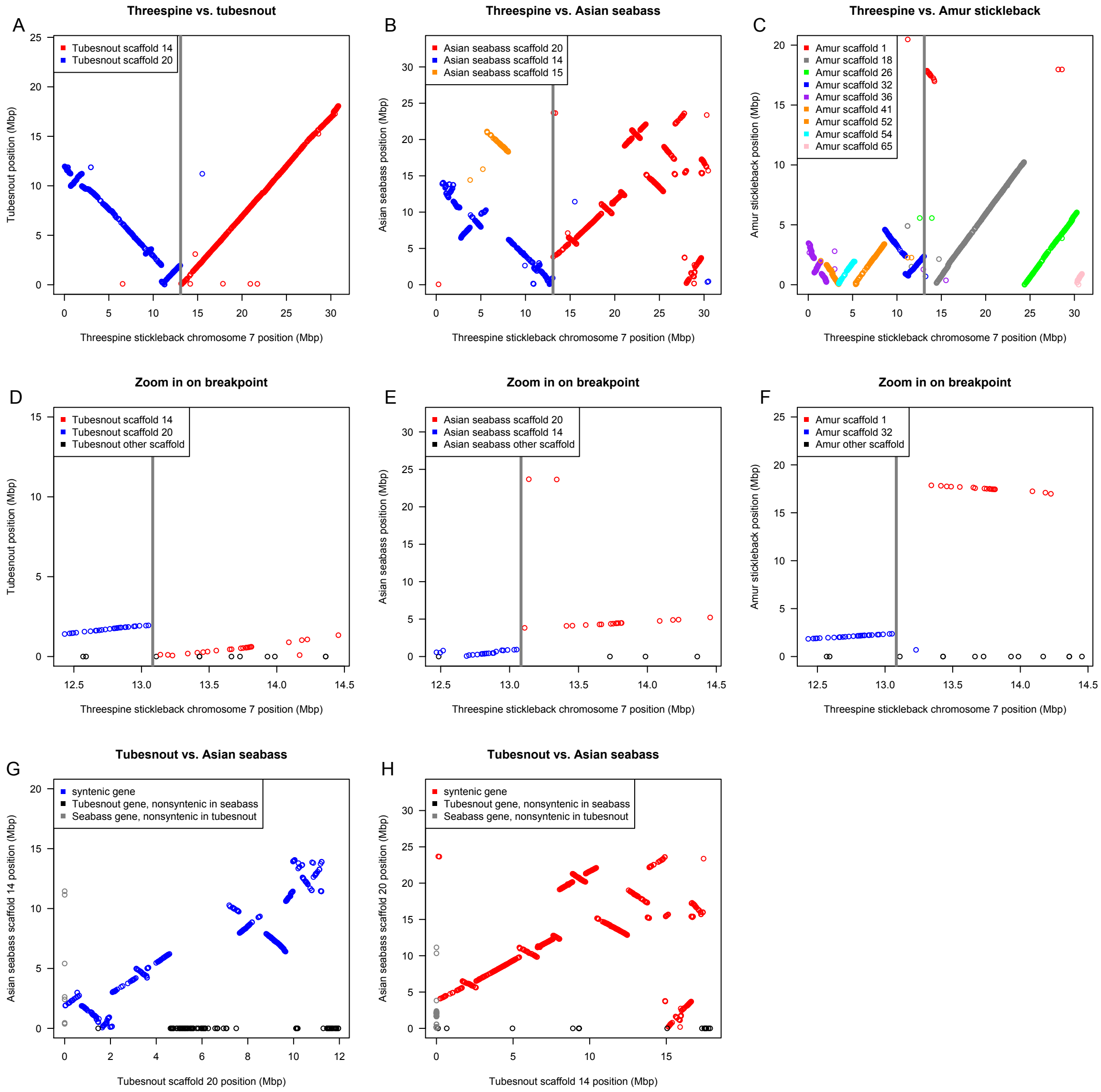

Figure S5. Patterns of synteny among scaffolds homologous to threespine stickleback chromosome VII. Each point represents the mapping of a single gene using method (2) that either supports the synteny (coloured points) or maps to some other scaffold in the assembly (black or grey; excluded from panels A-C for clarity). Vertical grey line marks the position of the mapping-breakpoint on threespine chromosome VII.

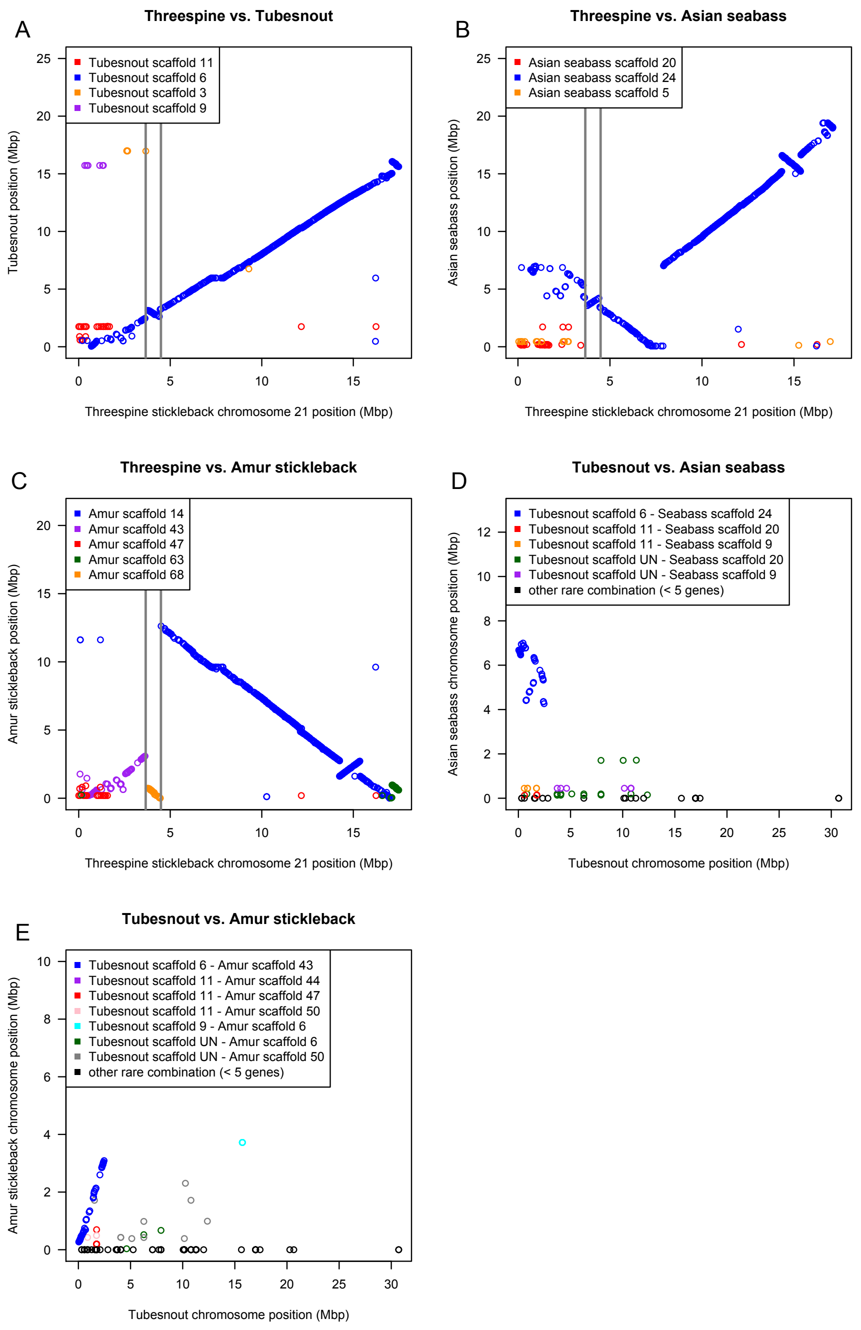

Figure S6. Patterns of synteny among scaffolds homologous to threespine stickleback chromosome XXI. Each point represents the mapping of a single gene using method (2) that either supports the synteny (coloured points) or maps to some other scaffold in the assembly (black or grey; excluded from panels A-C for clarity). Vertical grey lines added to illustrate the positioning of an apparent inversion (A & B) and likely case of under-assembly (C). Panels D & E show correspondence for the genes homologous to threespine stickleback Chr XXI in the rearranged region from 0 – 3.65 Mbp.

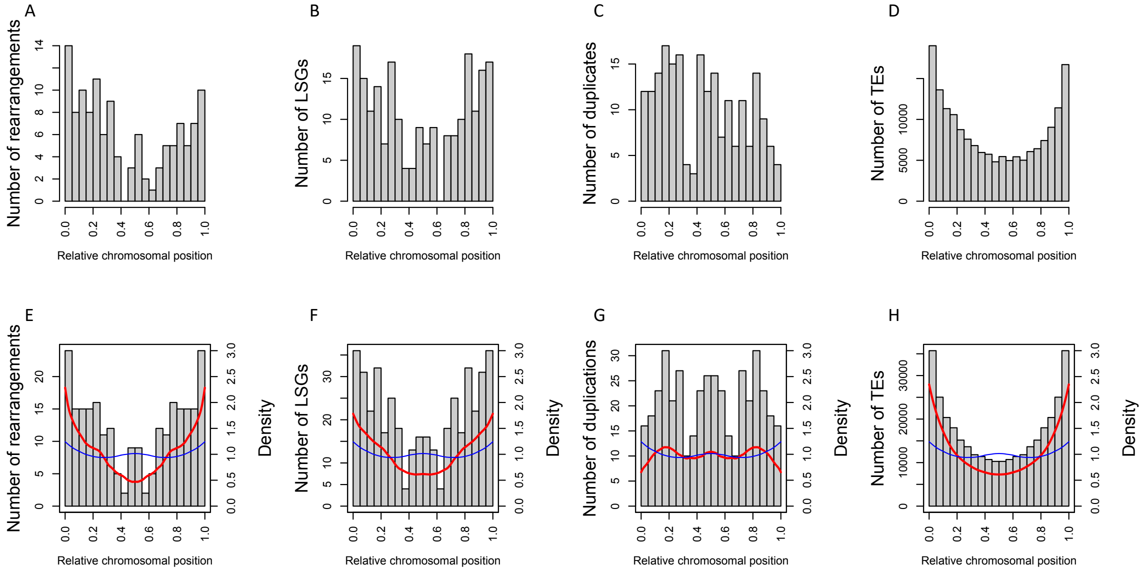

Figure S7. Density of rearrangements, Lineage-Specific Genes (LSGs), duplications, and Transposable Elements (TEs) by relative position for all chromosomes without macro-rearrangements (A-D) as well as the reflected density models fitted to this data (E-H). Red curves in E-H show the bounded density models fit using “boundarykernel” in R, while blue curves show gene density. As these models were used to test for enrichment of MGEEs in different regions of chromosomes, the density counts were calculated by excluding putative tandem duplicates within <1Mbp of each other.

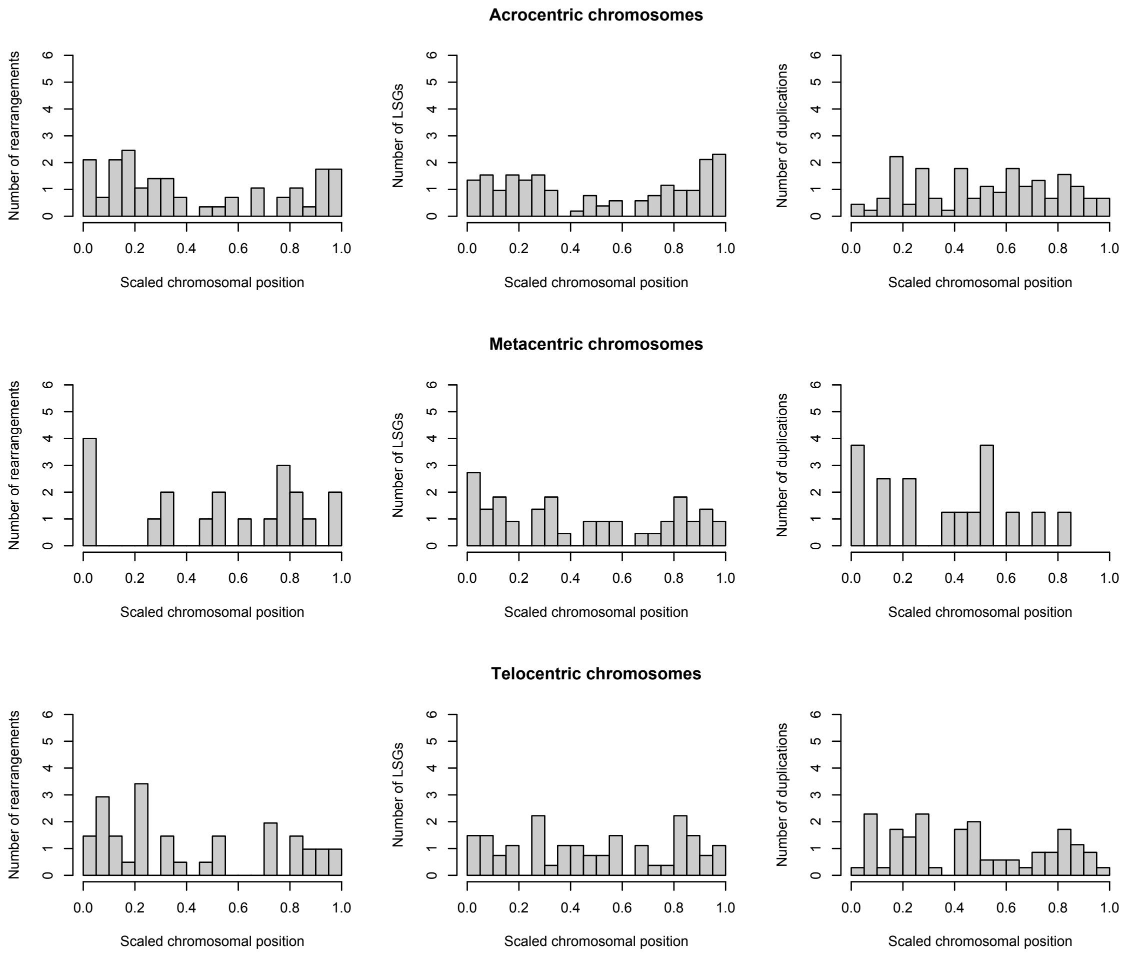

Figure S8. Chromosomal distribution of the three types of MGEE for three main chromosomal morphologies in threespine stickleback. Data excludes the X chromosome (Chr XIX) and the four chromosomes with macro-rearrangements. Morphologies taken from (Urton et al. 2011), with sub-metacentric and metacentric types grouped together.

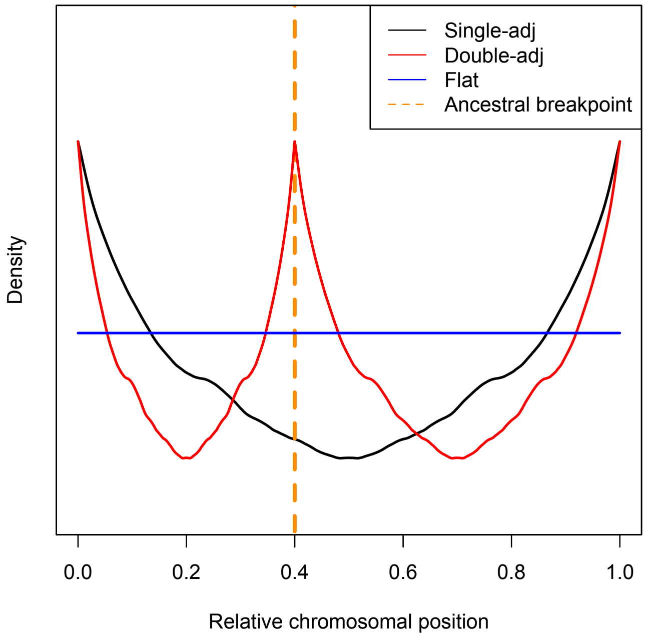

Figure S9. Three types of density models used for testing enrichment of MGEEs and TEs, with an example based on the density of micro-rearrangements in the non-rearranged chromosomes. The "flat" model is a uniform distribution, while both the "single-adj" and "double-adj" models are based on the density of event occurrence in the non-rearranged chromsomes, with rescaled and reflected positions as per Figure S7. The "double-adj" model accounts for the potential enrichment of events around the ancestral chromosomal breakpoints by fitting two scaled density models on either side of the breakpoints, while the "single-adj" model fits a single scaled density model without accounting for the breakpoint.

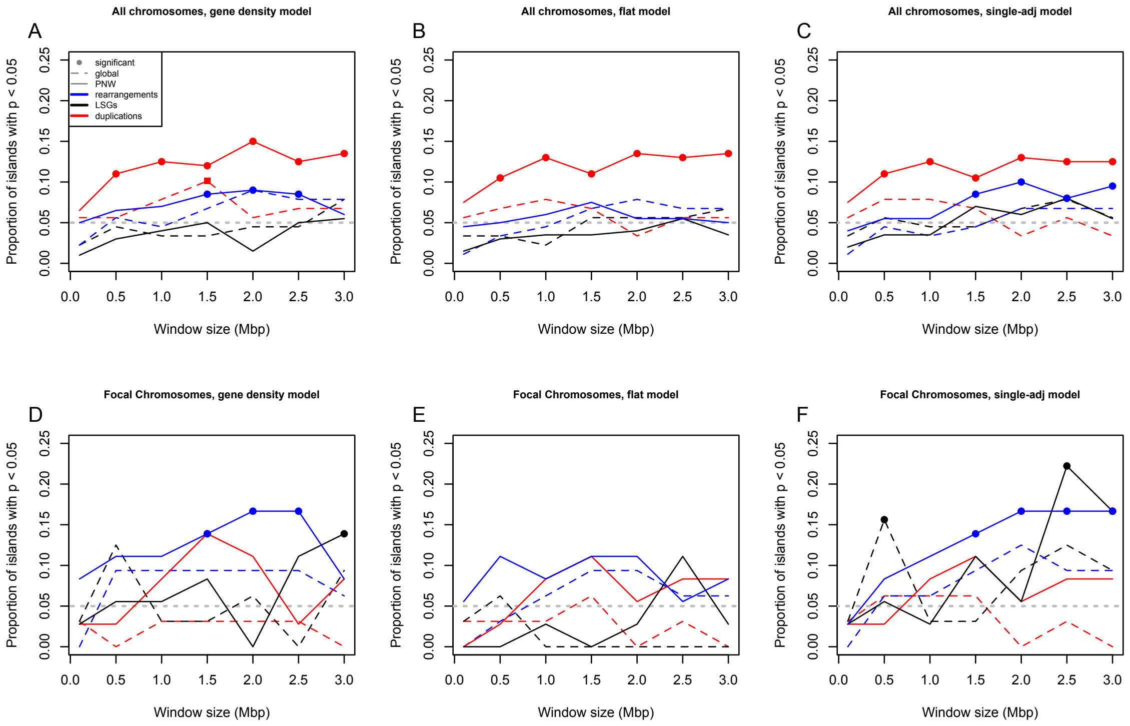

Figure S10. The proportion of genomic islands in the PNW and global sets of "ecopeaks" from Kingman et al. (2021) with significant enrichment of different types of MGEE. Enrichment analysis is shown for three null models, based on gene density (A, D), a uniform distribution (B, E), and MGEE occurrence, applied irrespective of macro-rearrangement breakpoints (C, F). Significance in the plot indicated by the filled dots occurs when the number of windows with $p <$ 0.05 exceeds the 95th percentile of a binomial distribution, with the null expectation of 5% indicated by a horizontal dashed grey line.

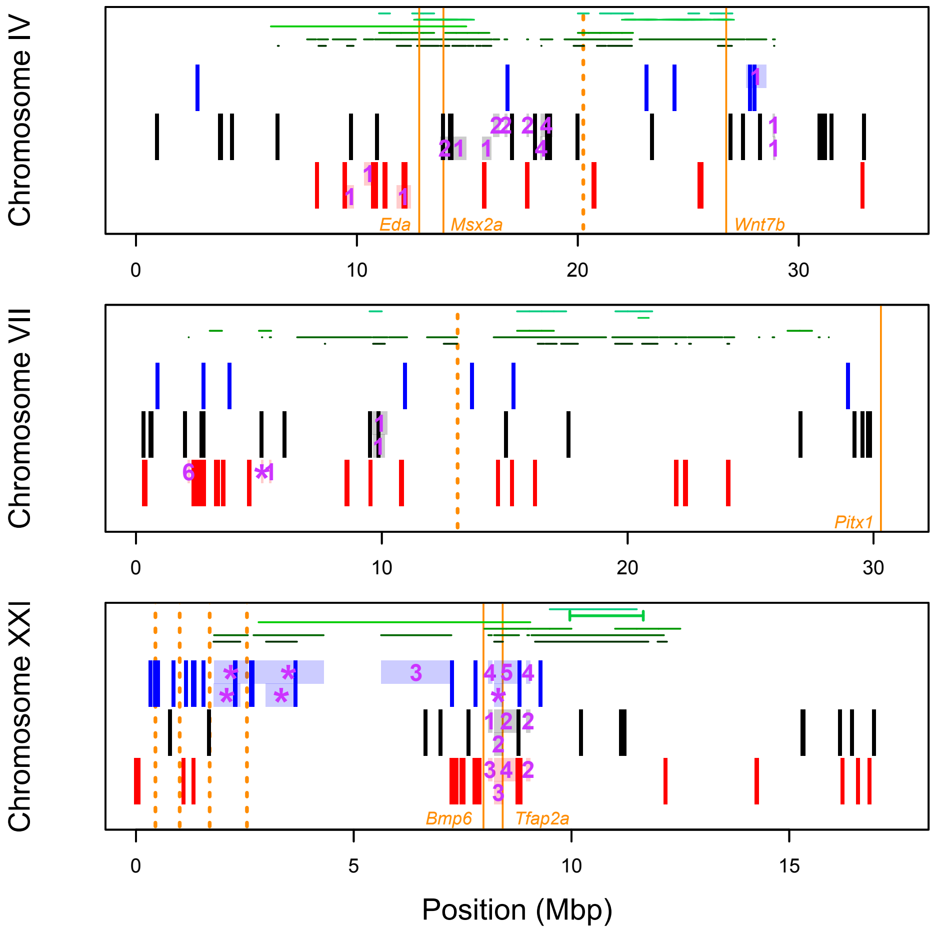

Figure S11. Chromosomal distribution of three types of Micro-Genomic Evolution Event (MGEE): micro-rearrangements (blue), Lineage Specific Genes (LSGs; black), and duplications (red) with enrichment testing under a null model where MGEEs occur in proportion to gene density. Shaded rectangles indicate regions that are significantly enriched for each type of MGEE ($p < 0.05$) around the Kingman PNW (above) and global (below) sets of genomic islands. Purple numbers indicate how many of the 7 window sizes were found to be significant, with "*" indicating an island that was significant following FDR correction across all islands tested ($q < 0.05$). The locations of candidate genes for local adaptation are shown with solid orange lines; orange dashed lines indicate the approximate location of breakpoints for ancestral macro-rearrangements. Lines along the top of each panel indicate positions where: mean F_{ST} between marine-freshwater populations from Samuk et al. (2017) falls in the top 5% of the distribution; extreme marine-freshwater divergence identified by Jones *et al*., (2012; with the inversion on ChrXXI identified as a bounded line); QTL identified by Miller et al. (2014); number of QTL from the meta-analysis of Peichel and Marques (Peichel and Marques 2017) falls into the top 5% of the distribution (dark green), and the PNW and global sets of genomic islands from Kingman et al (2021).

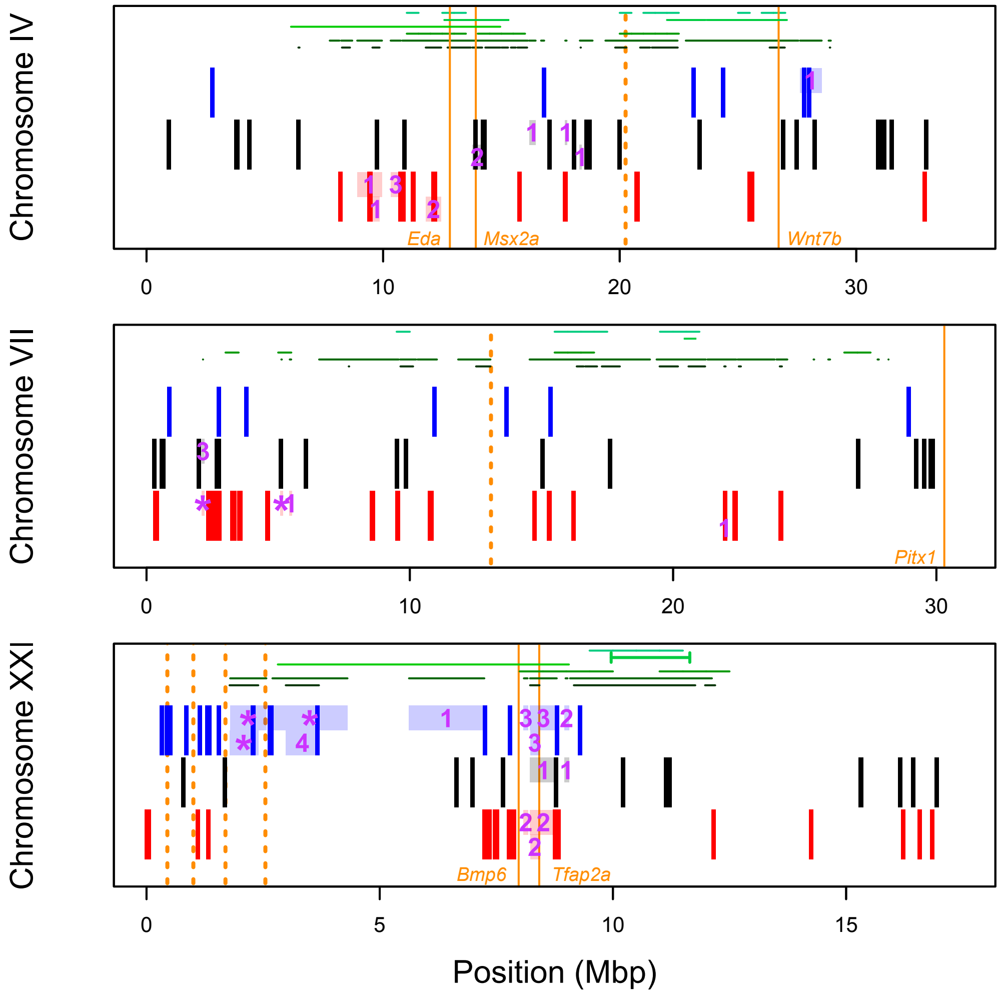

Figure S12. Chromosomal distribution of three types of Micro-Genomic Evolution Event (MGEE): micro-rearrangements (blue), Lineage Specific Genes (LSGs; black), and duplications (red) with enrichment testing under the "flat" null model where MGEEs occur following a uniform distribution.

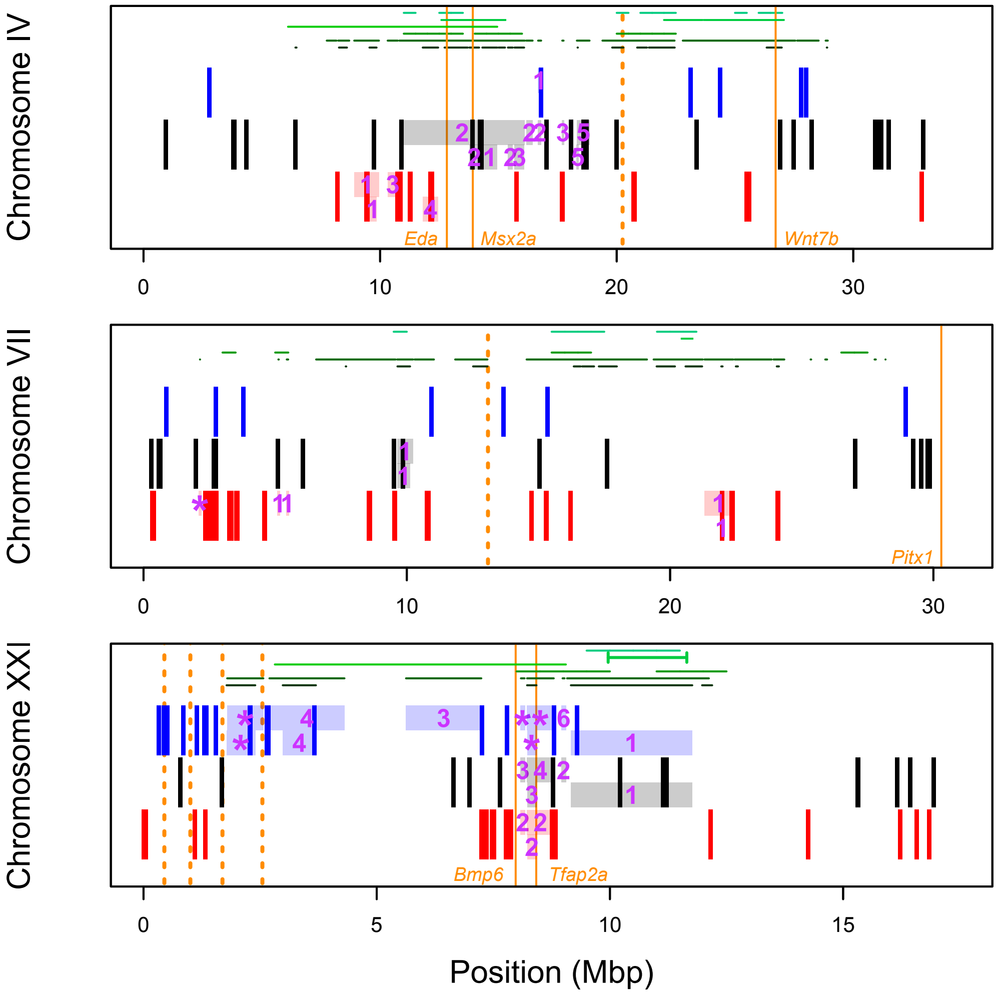

Figure S13. Chromosomal distribution of three types of Micro-Genomic Evolution Event (MGEE): micro-rearrangements (blue), Lineage Specific Genes (LSGs; black), and duplications (red) with enrichment testing under the “single-adj” null model where MGEEs occur following a their average density of occurrence, applied irrespective of macro-rearrangement breakpoints, from Figure S7.

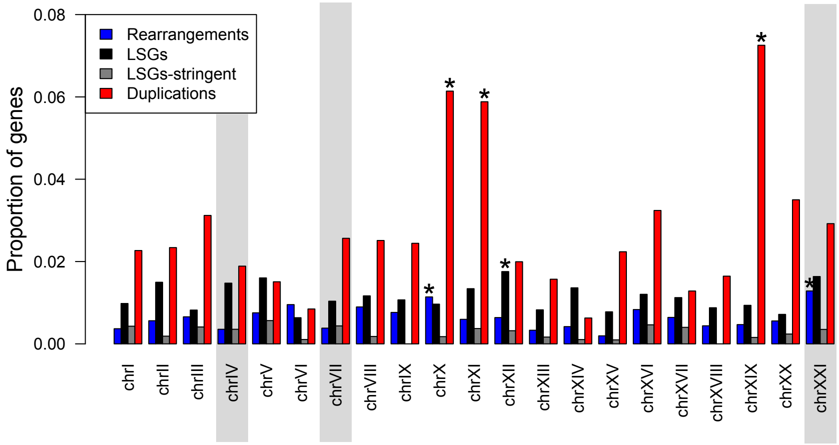

Figure S14. Proportion of genes on each chromosome that are either micro-rearrangements, Lineage Specific Genes (LSGs), or duplications. Chromosome-specific enrichment was assessed with a binomial test based on the genome-wide proportion of genes of each type of MGEE and the number of genes per chromosome, and cases with $p < 0.05$ are indicated by (*).

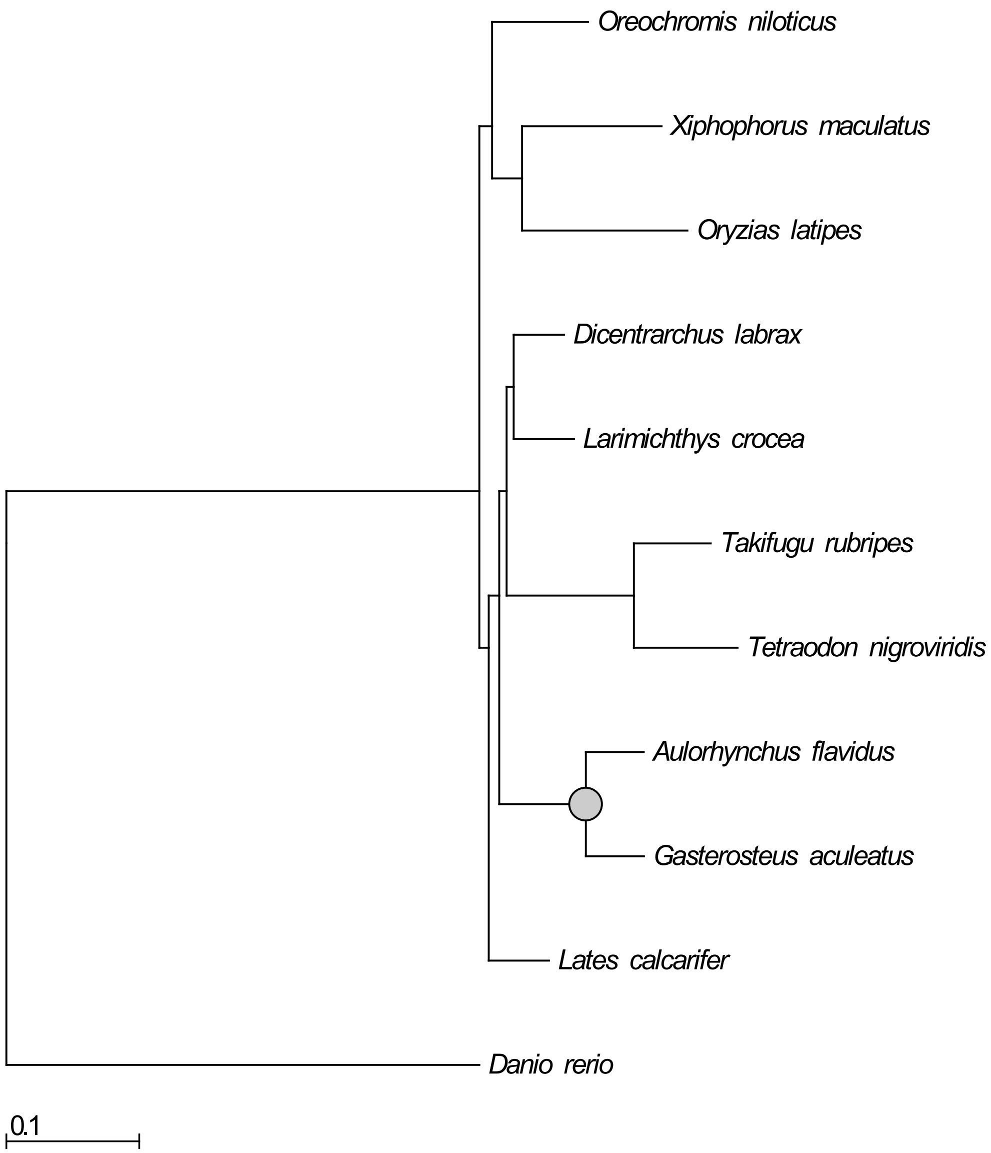

Figure S15: Maximum likelihood phylogenetic tree based on 2,504 genome-wide orthologs from 11 species. All nodes were supported by 100% of 50 performed bootstrap replicates. Zebrafish (*Danio rerio*) was used as outgroup for phylogenetic inference. The gray circle indicates the position of the threespine stickleback – tubesnout ancestor whose genome was reconstructed, using all eight additional teleosts and *D. rerio* as outgroup. The tree was visualized using Archaeopteryx (Han and Zmasek 2009).

Table S1. Lines of evidence used to identify chromosomes most strongly contributing to local adaptation in threespine stickleback for the study of rearrangements. Within each row, the 1st, 2nd, and 3rd ranked chromosome are highlighted in red, orange, and yellow (with ties labelled with the same colour). Note that the data from Hohenlohe et al. (2010) are included as a subset within Samuk et al. (2017), and the data from Miller et al. (2014) are included as a subset within Peichel and Marques (2017).

Evidence	ChrI	ChrIV	ChrVII	ChrXI	ChrXVI	ChrXX	ChrXXI	Reference
Average F_{ST} (marine-freshwater)	0.441 (6th)	0.491 (1st)	0.455 (3rd)	0.458 (2nd)	0.384 (16th)	0.440 (7th)	0.446 (4th)	Samuk et al. (2017), dataset shared by author
Average F_{ST} (marine-freshwater)	0.130 (6th)	0.160 (1st)	0.149 (3rd)	0.144 (4th)	0.098 (16th)	0.137 (5th)	0.159 (2nd)	Hohenlohe et al. (2010), dataset shared by author
Regions of extreme marine-freshwater divergence	Yes	Yes	Yes	No	No	No	No	Jones et al. (2012); Supplementary Data, Figure 6
Inversion associated with marine-freshwater	Yes	No	No	Yes	No	No	Yes	Jones et al. (2012); Figure 3, red arrows
Number of QTL	28	65	28	9	12	42	26	Miller et al. (2014), Figure 3
Significant enrichment of QTL	No	$p < 0.001$	No	No	No	$p < 0.05$	$p < 0.001$	Miller et al. (2014), Figure 6
Trait types showing significant enrichment of QTLs	0	3	1	0	1	1	3	Peichel and Marques (2017), Figure 3
Mean number of QTL per window	4.7 (6th)	8.4 (1st)	5.3 (4th)	1.9 (13th)	5.1 (5th)	5.6 (3rd)	8.1 (2nd)	Peichel and Marques (2017), Table S1

Table S2. Percentage of genes that are syntenic between threespine stickleback and tubesnout chromosomes that have no macro-rearrangements, as well as the number of genes that are syntenic to the second most syntenic tubesnout chromosome ("secondary-syntenic genes").

Threespine stickleback chromosome	Tubesnout scaffold	Number of syntenic genes	Number of non-syntenic genes	Percent syntenic genes	Number of secondary-syntenic genes
chrII	22	1027	67	0.939	9
chrIII	18	1159	52	0.957	7
chrV	16	958	64	0.937	8
chrVI	11	910	51	0.947	7
chrVIII	2	1048	52	0.953	5
chrIX	8	1201	69	0.946	8
chrX	7	1032	95	0.916	22
chrXI	17	1239	59	0.955	9
chrXII	10	1145	55	0.954	6
chrXIII	1	1160	51	0.958	8
chrXIV	12	879	33	0.964	5
chrXV	9	1011	36	0.966	4
chrXVI	3	957	91	0.913	10
chrXVII	4	1203	43	0.965	4
chrXVIII	19	830	41	0.953	5
chrXIX	23	1230	59	0.954	10
chrXX	13	1200	60	0.952	10
chrUn	1	100	747	0.118	95

Table S3. Enrichment of the three types of Micro-Genome Evolution Events (MGEEs) around ancestral chromosomal breakpoints for the four chromosomes with macro-rearrangements, as represented by the observed / expected value under the three null density models. Significant *p*-values are shaded in light grey ($0.01 < p < 0.05$) and dark grey ($p < 0.01$; note: no correction for multiple contrasts); models reported in the main text are indicated by (*). Density models indicated by the test types are shown in Figure S9. Note that the double-adj model was not fit to chrXXI due to difficulty of reconstructing the complex rearrangement.

			Distance from chromosomal breakpoint (kb)						
Chromosome	Test type	MGEE	100	500	1000	1500	2000	2500	3000
chrI	double-adj*	duplication	0.0	1.9	2.6	1.6	1.5	1.2	1.0
chrI	flat	duplication	0.0	1.8	2.6	1.8	1.7	1.4	1.2
chrI	single-adj	duplication	0.0	1.8	2.7	1.8	1.8	1.4	1.2
chrIV	double-adj*	duplication	0.0	3.7	1.7	1.1	0.8	0.6	0.9
chrIV	flat	duplication	0.0	2.9	1.5	1.0	0.7	0.6	1.0
chrIV	single-adj	duplication	0.0	3.1	1.5	1.0	0.7	0.6	1.0
chrVII	double-adj*	duplication	0.0	0.0	0.0	0.0	0.4	0.9	0.7
chrVII	flat	duplication	0.0	0.0	0.0	0.0	0.4	0.8	0.7
chrVII	single-adj	duplication	0.0	0.0	0.0	0.0	0.4	0.9	0.7
chrXXI	flat	duplication	0.7	1.0	0.9	0.8	0.7	0.6	0.6
chrXXI	single-adj*	duplication	0.7	1.1	0.9	0.8	0.7	0.6	0.6
chrI	double-adj*	LSG	0.0	0.0	0.0	0.0	0.3	0.2	0.2
chrI	flat	LSG	0.0	0.0	0.0	0.0	0.5	0.4	0.3
chrI	single-adj	LSG	0.0	0.0	0.0	0.0	0.6	0.5	0.4
chrIV	double-adj*	LSG	0.0	0.8	0.4	0.3	0.7	0.7	0.6
chrIV	flat	LSG	0.0	1.4	0.7	0.5	1.0	1.1	0.9
chrIV	single-adj	LSG	0.0	2.3	1.1	0.7	1.7	1.8	1.5

chrVII	double-adj*	LSG	0.0	0.0	0.0	0.0	0.3	0.2	0.2
chrVII	flat	LSG	0.0	0.0	0.0	0.0	0.4	0.3	0.3
chrVII	single-adj	LSG	0.0	0.0	0.0	0.0	0.7	0.5	0.4
chrXXI	flat	LSG	1.1	0.8	0.7	0.6	0.5	0.5	0.5
chrXXI	single-adj*	LSG	0.8	0.6	0.5	0.5	0.4	0.4	0.4
chrI	double-adj*	rearrangement	9.2	4.3	3.7	2.7	2.9	2.4	2.6
chrI	flat	rearrangement	24.3	9.9	7.5	5.0	4.9	4.0	4.1
chrI	single-adj	rearrangement	31.3	12.7	9.4	6.1	6.1	4.8	5.1
chrIV	double-adj*	rearrangement	0.0	0.0	0.0	0.0	0.0	0.0	0.6
chrIV	flat	rearrangement	0.0	0.0	0.0	0.0	0.0	0.0	1.0
chrIV	single-adj	rearrangement	0.0	0.0	0.0	0.0	0.0	0.0	1.7
chrVII	double-adj*	rearrangement	0.0	0.0	1.2	0.9	0.7	1.7	1.5
chrVII	flat	rearrangement	0.0	0.0	2.3	1.5	1.1	2.6	2.2
chrVII	single-adj	rearrangement	0.0	0.0	4.2	2.8	2.1	4.9	4.1
chrXXI	flat	rearrangement	4.2	3.1	2.7	2.7	2.4	2.2	2.0
chrXXI	single-adj*	rearrangement	2.8	2.1	1.9	2.0	1.8	1.7	1.6

Table S4. Enrichment of the three types of Micro-Genome Evolution Events (MGEEs) around candidate genes, as represented by the observed / expected value under the three null density models. Significant *p*-values are shaded in light grey ($0.01 < p < 0.05$) and dark grey ($p < 0.01$; note: no correction for multiple contrasts). Density models indicated by the test types are shown in Figure S9. Note that the double-adj model was not fit to chrXXI due to difficulty of reconstructing the complex rearrangement.

				Distance from candidate gene (kb)						
Chromosome	Candidate gene	Test type	MGEE	100	500	1000	1500	2000	2500	3000
chrIV	eda/msx2a	double-adj	duplication	0	0	3.5	2.6	3.4	3.3	2.8
chrIV	eda/msx2a	flat	duplication	0	0	2.8	2.1	2.8	2.8	2.4
chrIV	eda/msx2a	single-adj	duplication	0	0	2.9	2.2	2.9	2.9	2.5
chrIV	wnt7b	double-adj	duplication	0	0	0	0.8	0.6	0.5	0.4
chrIV	wnt7b	flat	duplication	0	0	0	0.9	0.7	0.6	0.5
chrIV	wnt7b	single-adj	duplication	0	0	0	0.8	0.6	0.5	0.4
chrVII	pitx1	double-adj	duplication	0	0	0	0	0	0	0
chrVII	pitx1	flat	duplication	0	0	0	0	0	0	0
chrVII	pitx1	single-adj	duplication	0	0	0	0	0	0	0
chrXXI	bmp6/tfap2a	flat	duplication	0	4.4	3.3	2.3	1.8	1.5	1.2
chrXXI	bmp6/tfap2a	single-adj	duplication	0	4.3	3.2	2.3	1.8	1.5	1.3
chrIV	eda/msx2a	double-adj	LSG	1.8	3.3	2.2	1.6	1.7	1.4	1.2
chrIV	eda/msx2a	flat	LSG	1	1.9	1.3	1	1.1	0.9	0.8
chrIV	eda/msx2a	single-adj	LSG	1.6	3	2.1	1.6	1.7	1.4	1.2
chrIV	wnt7b	double-adj	LSG	0	1.9	1.9	1.2	1.4	1.1	0.8
chrIV	wnt7b	flat	LSG	0	1.4	1.4	0.9	1	0.8	0.7
chrIV	wnt7b	single-adj	LSG	0	1.3	1.3	0.9	1	0.8	0.7
chrVII	pitx1	double-adj*	LSG	0	1.2	2.4	2.4	2	1.7	1.5

chrVII	pitx1	flat	LSG	0	1.6	3.1	3.1	2.5	2.1	1.8
chrVII	pitx1	single-adj	LSG	0	1	1.9	2	1.6	1.4	1.2
chrXXI	bmp6/tfap2a	flat	LSG	0	1.7	1.5	1.4	1.4	1.1	1.4
chrXXI	bmp6/tfap2a	single-adj	LSG	0	2.9	2.5	2.4	2.3	1.8	2.1
chrIV	eda/msx2a	double-adj	rearrangement	0	0	0	0	0	0	1.3
chrIV	eda/msx2a	Flat	rearrangement	0	0	0	0	0	0	0.8
chrIV	eda/msx2a	single-adj	rearrangement	0	0	0	0	0	0	1.3
chrIV	wnt7b	double-adj	rearrangement	0	0	0	6.2	4.3	4.9	3.7
chrIV	wnt7b	Flat	rearrangement	0	0	0	3.8	2.9	3.4	2.9
chrIV	wnt7b	single-adj	rearrangement	0	0	0	3.6	2.7	3.3	2.8
chrVII	pitx1	double-adj	rearrangement	0	0	0	1.5	1.3	1.1	1
chrVII	pitx1	Flat	rearrangement	0	0	0	2.1	1.7	1.4	1.2
chrVII	pitx1	single-adj	rearrangement	0	0	0	1.2	1	0.9	0.8
chrXXI	bmp6/tfap2a	Flat	rearrangement	0	2.2	2.6	1.9	1.4	1.2	1
chrXXI	bmp6/tfap2a	single-adj	rearrangement	0	4.7	5.5	3.8	2.8	2.2	1.7

Table S5. Results of GO enrichment analysis for significant hits among the genes involved in duplications or micro-rearrangements.

Type	Source	Term name	Term id	FDR-adjusted p-value	Term size	Query size	Intersection size	Effective domain size
duplicate	GO:MF	G protein-coupled receptor activity	GO:0004930	0.0008	619	135	20	13744
duplicate	GO:MF	olfactory receptor activity	GO:0004984	0.0033	77	135	7	13744
duplicate	GO:BP	G protein-coupled receptor signaling pathway	GO:0007186	0.0019	720	104	20	12055
duplicate	GO:BP	detection of chemical stimulus involved in sensory perception of smell	GO:0050911	0.0029	77	104	7	12055
duplicate	GO:BP	detection of chemical stimulus	GO:0009593	0.0031	78	104	7	12055
duplicate	GO:BP	detection of chemical stimulus involved in sensory perception	GO:0050907	0.0031	78	104	7	12055
duplicate	GO:BP	sensory perception of smell	GO:0007608	0.0034	79	104	7	12055
duplicate	GO:BP	sensory perception of chemical stimulus	GO:0007606	0.0052	84	104	7	12055
duplicate	GO:BP	detection of stimulus	GO:0051606	0.0114	131	104	8	12055
duplicate	GO:BP	detection of stimulus involved in sensory perception	GO:0050906	0.0125	96	104	7	12055
duplicate	GO:BP	gas transport	GO:0015669	0.0154	20	104	4	12055
duplicate	GO:CC	hemoglobin complex	GO:0005833	0.0395	13	113	3	11288
duplicate	HP	Recurrent fever	HP:0001954	0.0111	82	28	6	4490
Rearrangement	GO:CC	axonemal dynein complex	GO:0005858	0.0061	5	29	2	11288
Rearrangement	GO:CC	dynein complex	GO:0030286	0.0062	31	29	3	11288
Rearrangement	GO:CC	cilium	GO:0005929	0.0166	111	29	4	11288
Rearrangement	GO:CC	microtubule associated complex	GO:0005875	0.0331	54	29	3	11288

Table S6. Transposable Element (TE) enrichment around ancestral chromosomal breakpoints for the four chromosomes with macro-rearrangements, as represented by the observed / expected value under the three null density models. Significant *p*-values ($p < 0.05$) are shaded in grey; models reported in the main text are indicated by (*). Density models indicated by the test types are shown in Figure S9. Note that the double-adj model was not fit to chrXXI due to difficulty of reconstructing the complex rearrangement.

		Distance from chromosomal breakpoint (kb)						
Chromosome	Test type	100	500	1000	1500	2000	2500	3000
chrI	double-adj*	1.1	1.1	1.2	1.2	1.1	1.1	1.2
chrI	flat	2.9	2.7	2.5	2.4	2.1	1.9	1.8
chrI	single-adj	3.9	3.8	3.4	3.2	2.8	2.6	2.5
chrIV	double-adj*	0.8	1.0	1.0	1.0	1.1	1.2	1.3
chrIV	flat	2.0	2.2	2.0	1.9	1.9	1.9	2.0
chrIV	single-adj	3.1	3.7	3.2	3.1	3.1	3.1	3.2
chrVII	double-adj*	1.1	1.1	1.1	1.3	1.3	1.4	1.3
chrVII	flat	2.5	2.4	2.2	2.3	2.3	2.1	2.0
chrVII	single-adj	3.9	3.9	3.7	3.8	3.7	3.5	3.2
chrXXI	flat	2.0	2.1	2.1	2.0	2.0	1.9	1.8
chrXXI	single-adj*	1.3	1.4	1.5	1.5	1.5	1.5	1.5

Supplementary References:

Altenhoff AM, Škunca N, Glover N, Train C-M, Sueki A, Piližota I, Gori K, Tomiczek B, Müller S, Redestig H, et al. 2015. The OMA orthology database in 2015: function predictions, better plant support, synteny view and other improvements. *Nucleic Acids Research* 43:D240–D249.

Glazer AM, Killingbeck EE, Mitros T, Rokhsar DS, Miller CT. 2015. Genome Assembly Improvement and Mapping Convergently Evolved Skeletal Traits in Sticklebacks with Genotyping-by-Sequencing. *G3* 5:1463–1472.

Han MV, Zmasek CM. 2009. phyloXML: XML for evolutionary biology and comparative genomics. *BMC Bioinformatics* 10:356.

Huerta-Cepas J, Forslund K, Coelho LP, Szklarczyk D, Jensen LJ, von Mering C, Bork P. 2017. Fast Genome-Wide Functional Annotation through Orthology Assignment by eggNOG-Mapper. *Molecular Biology and Evolution* 34:2115–2122.

Jones FC, Brown C, Pemberton JM, Braithwaite VA. 2006. Reproductive isolation in a threespine stickleback hybrid zone. *Journal of Evolutionary Biology*. 19:531-544.

Jones FC, Grabherr MG, Chan YF, Russell P, Mauceli E, Johnson J, Swofford R, Pirun M, Zody MC, et al. 2012. The genomic basis of adaptive evolution in threespine sticklebacks. *Nature* 484:55–61

Katoh K. 2002. MAFFT: a novel method for rapid multiple sequence alignment based on fast Fourier transform. *Nucleic Acids Research* 30:3059–3066.

Kawase J, Aoki J, Araki K. 2018. Constructing a "Chromonome" of Yellowtail (*Seriola quinqueradiata*) for Comparative Analysis of Chromosomal Rearrangements. *J. Genomics* 6:9–19.

McCairns RJS, Bernatchez L. Adaptive divergence between freshwater and marine sticklebacks: insights into the role of phenotypic plasticity from an integrated analysis of candidate gene expression. *Evolution* 65:1029-1047.

Miller CT, Glazer AM, Summers BR, Blackman BK, Norman AR, Shapiro MD, Cole BL, Peichel CL, Schluter D, Kingsley DM. 2014. Modular Skeletal Evolution in Sticklebacks Is Controlled by Additive and Clustered Quantitative Trait Loci. *Genetics* 197:405–420.

Near TJ, Dornburg A, Eytan RI, Keck BP, Smith WL, Kuhn KL, Moore JA, Price SA, Burbrink FT, Friedman M, et al. 2013. Phylogeny and tempo of diversification in the superradiation of spiny-rayed fishes. *Proceedings of the National Academy of Sciences* 110:12738–12743.

Peichel CL, Marques DA. 2017. The genetic and molecular architecture of phenotypic diversity in sticklebacks. *Phil. Trans. R. Soc. B* 372:20150486.

Peichel CL, Sullivan ST, Liachko I, White MA. 2017. Improvement of the Threespine Stickleback Genome Using a Hi-C-Based Proximity-Guided Assembly. *Journal of Heredity* 108:693–700.

Rastas P, Calboli FCF, Guo B, Shikano T, Merilä J. 2016. Construction of Ultradense Linkage Maps with Lep-MAP2: Stickleback F_2 Recombinant Crosses as an Example. *Genome Biol Evol* 8:78–93.

Raudvere U, Kolberg L, Kuzmin I, Arak T, Adler P, Peterson H, Vilo J. 2019. g:Profiler: a web server for functional enrichment analysis and conversions of gene lists (2019 update). *Nucleic Acids Research* 47:W191–W198.

Reimand J, Kull M, Peterson H, Hansen J, Vilo J. 2007. g:Profiler—a web-based toolset for functional profiling of gene lists from large-scale experiments. *Nucleic Acids Research* 35:W193–W200.

Samuk K, Owens GL, Delmore KE, Miller SE, Rennison DJ, Schluter D. 2017. Gene flow and selection interact to promote adaptive divergence in regions of low recombination. *Mol Ecol* 26:4378–4390.

Stamatakis A. 2014. RAxML version 8: a tool for phylogenetic analysis and post-analysis of large phylogenies. *Bioinformatics* 30:1312–1313.

Tine M, Kuhl H, Gagnaire P-A, Louro B, Desmarais E, Martins RST, Hecht J, Knaust F, Belkhir K, Klages S, et al. 2014. European sea bass genome and its variation provide insights into adaptation to euryhalinity and speciation. *Nat Commun* 5:5770.

Urton JR, McCann SR, Peichel CL. 2011. Karyotype Differentiation between Two Stickleback Species (Gasterosteidae). *Cytogenet Genome Res* 135:150–159.

Varadharajan S, Rastas P, Löytynoja A, Matschiner M, Calboli Federico C F, Guo B, Nederbragt AJ, Jakobsen KS, Merilä J. 2019. A high-quality assembly of the nine-spined stickleback (Pungitius pungitius) genome. *Genome Biology and Evolution* 11:3291-3308.

Vij S, Kuhl H, Kuznetsova IS, Komissarov A, Yurchenko AA, Heusden PV, Singh S, Thevasagayam NM, Prakki SRS, Purushothaman K, et al. 2016. Chromosomal-Level Assembly of the Asian Seabass Genome Using Long Sequence Reads and Multi-layered Scaffolding. *PLOS Genetics*:35.
